# From Dual Targeting to Clinical Validation: A Translational Framework for Bispecific Antibody–Drug Conjugates

**DOI:** 10.3390/ijms27198748

**Published:** 2026-09-30

**Authors:** Jun Kim, Seounghun Kang

**Affiliations:** 1 Department of Chemistry, Graduate School, Soongsil University, Seoul 06978, Republic of Korea; seallingfx@gmail.com; 2 AI-Bio Convergence Research Institute, Soongsil University, Seoul 06978, Republic of Korea

**Keywords:** bispecific antibody–drug conjugate, dual-antigen targeting, biparatopic ADC, target-pair biology, antibody engineering, drug delivery, translational development, biomarker, therapeutic index, clinical validation

## Abstract

Bispecific antibody–drug conjugates (bsADCs) are being developed to address limitations of conventional ADCs, including heterogeneous antigen expression, insufficient tumor–normal discrimination, and suboptimal intracellular trafficking. This review evaluates bsADCs from a preclinical-to-clinical translational perspective and defines the evidence needed to justify the added complexity of bispecific design. A structured search of PubMed/MEDLINE, Embase, Web of Science, Scopus, clinical-trial registries, and major oncology conference proceedings was conducted through 29 July 2026. Dual-antigen bsADCs were classified as coverage-oriented, co-expression-dependent, trafficking-assisted, or multifunctional, while biparatopic ADCs were considered mechanistically related comparators. Dual recognition can broaden tumor coverage, enhance avidity-dependent selectivity, improve intracellular trafficking, or add biological functions. These effects depend on target-pair biology, molecular format, valency, epitope geometry, arm affinity, linker–payload properties, drug-to-antibody ratio, pharmacokinetics, normal-tissue exposure, manufacturability, and biomarker feasibility. Clinically, izalontamab brengitecan supports the feasibility of selected architectures, whereas MEDI4276 and TQB2102 illustrate how complete molecular design shapes therapeutic index. We propose an integrated translational framework and functional design parsimony, defined as selecting the least complex ADC architecture capable of achieving the intended biological and translational objective. Bispecificity should be used only when it provides a demonstrable advantage over a simpler ADC design.

## 1. Introduction

Antibody–drug conjugates (ADCs) have become an established therapeutic modality that combines the target selectivity of monoclonal antibodies with the cytotoxic potency of small-molecule payloads. Advances in antigen selection, antibody engineering, conjugation chemistry, linker stability, and payload design have substantially expanded their clinical application across hematologic malignancies and solid tumors. Nevertheless, an ADC does not behave simply as the sum of its individual components. Its efficacy, pharmacokinetics, and toxicity emerge from the coordinated properties of the target antigen, antibody, linker, payload, conjugation site, and drug-to-antibody ratio. Accordingly, the development of an effective ADC requires simultaneous optimization of tumor targeting, intracellular payload delivery, systemic stability, and tolerability [1,2,3].

These requirements are particularly difficult to satisfy in solid tumors. An ideal ADC target would be highly and uniformly expressed on malignant cells, minimally present in normal tissues, readily accessible to circulating antibodies, and capable of efficient internalization and lysosomal trafficking. However, few clinically relevant antigens possess all of these characteristics. Target expression often varies across patients, between primary and metastatic lesions, and among individual cells within the same tumor. Such heterogeneity can produce uneven intratumoral payload exposure and allow antigen-low or antigen-negative subpopulations to survive treatment. Therapeutic pressure may further promote antigen downregulation or loss, while alterations in receptor trafficking, lysosomal processing, drug-efflux transporters, and payload sensitivity can contribute to acquired resistance [4,5].

Increasing antibody affinity or payload potency alone does not necessarily overcome these limitations. Excessively high affinity can restrict tumor penetration through a binding-site barrier, whereas highly membrane-permeable payloads may extend cytotoxicity to neighboring antigen-low cells at the cost of broader normal-tissue exposure. In addition, antigens with broad tumor prevalence are frequently expressed to some extent in essential normal tissues, creating a risk of on-target, off-tumor toxicity. The central challenge in ADC design is therefore not merely to maximize tumor-cell killing, but to achieve a favorable balance among antigen coverage, tissue penetration, intracellular processing, systemic stability, and therapeutic index [6,7].

Bispecific antibody–drug conjugates (bsADCs) have emerged as a strategy to address several of these constraints. In this review, bsADCs primarily refer to ADCs whose antibody component recognizes two distinct antigens. Biparatopic ADCs, which recognize two non-overlapping epitopes on the same antigen, are discussed as structurally distinct but mechanistically overlapping comparators within a broader continuum of multivalent receptor engagement. Through dual recognition, bsADCs may broaden tumor coverage, enhance binding on dual-positive cells, alter receptor organization and internalization, or use one antigen to redirect another toward a more productive intracellular trafficking pathway. Because both binding specificities are incorporated into a single conjugated molecule, bsADCs may generate spatial and functional interactions that cannot be reproduced by the simple co-administration of two monospecific ADCs [8,9].

**Figure 1:**
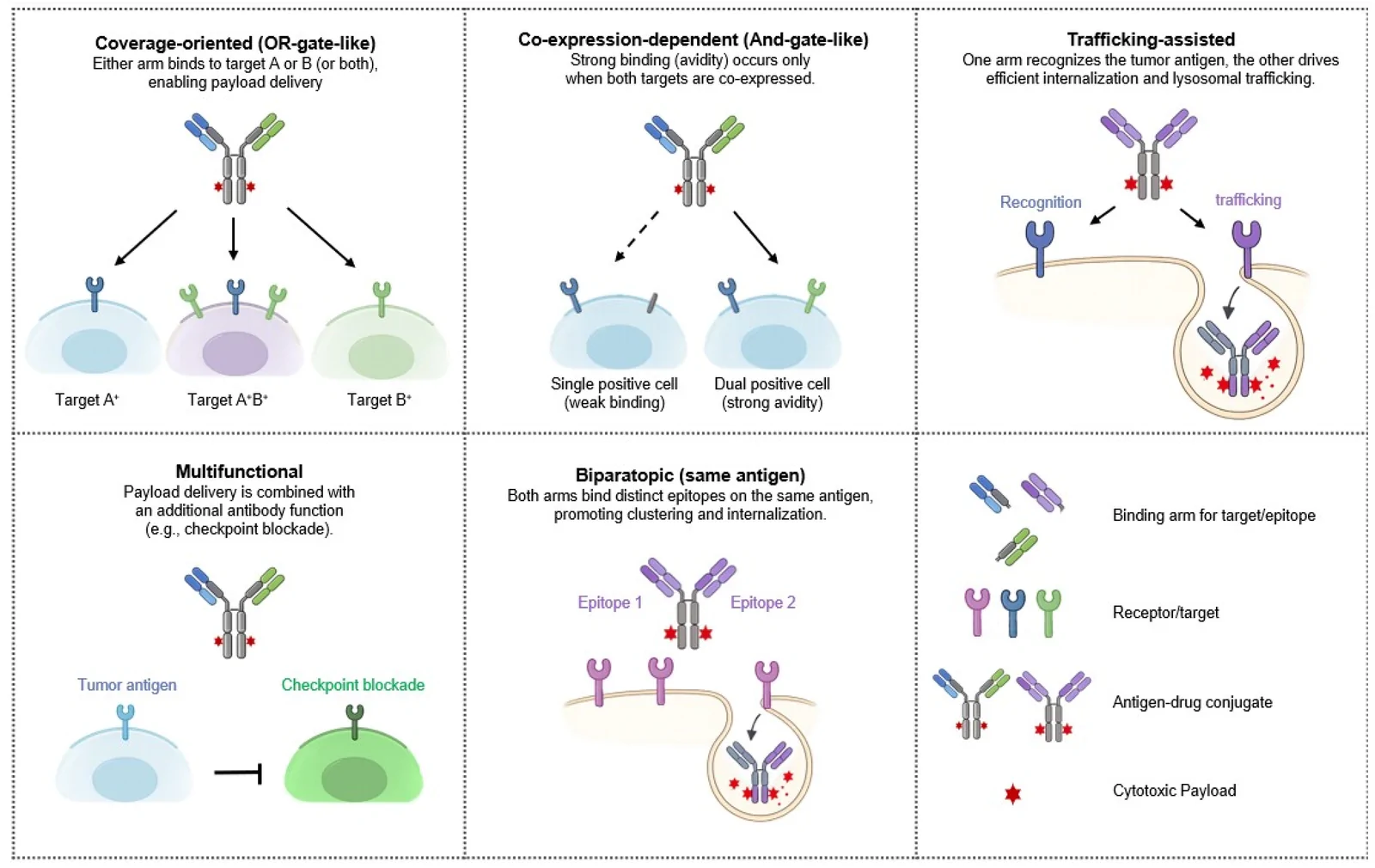
Functional design strategies of bispecific antibody–drug conjugates. Schematic overview of the principal functional strategies of bispecific antibody–drug conjugates (bsADCs). Coverage-oriented (OR-gate-like) bsADCs independently engage either target, allowing payload delivery to target A-positive, target B-positive, or dual-positive tumor-cell populations. Co-expression-dependent (AND-gate-like) designs exploit cross-arm avidity to preferentially bind and deliver payload to dual-positive cells, while binding to single-positive cells remains weaker. Trafficking-assisted bsADCs couple recognition of a tumor-associated antigen to a second receptor that promotes productive internalization and lysosomal routing of the conjugate. Multifunctional bsADCs combine cytotoxic payload delivery with an additional antibody-mediated activity, such as receptor or immune-checkpoint blockade. Biparatopic ADCs are shown as mechanistically related comparators within a broader continuum of multivalent receptor engagement. By binding two non-overlapping epitopes on the same antigen, they may promote intra- or intermolecular engagement, receptor crosslinking, higher-order receptor organization, internalization, or altered trafficking depending on epitope geometry and molecular format. Colored antibody arms represent distinct target or epitope specificities, cell-surface receptor symbols represent the corresponding targets, and red stars represent cytotoxic payloads. Solid arrows indicate productive binding or trafficking, whereas the dashed arrow in the co-expression-dependent panel denotes weaker binding to a single-positive cell. The blockade symbol in the multifunctional panel represents an additional non-cytotoxic antibody-mediated function. Payload placement is schematic and does not indicate a universal conjugation site.

The biological rationale for dual-antigen targeting can be broadly divided into coverage-oriented and selectivity-oriented strategies. In coverage-oriented designs, binding to either antigen enables engagement of phenotypically heterogeneous tumor-cell populations, potentially reducing escape associated with variable or dynamically changing antigen expression. This behavior is sometimes described as “OR-gate-like” targeting. In selectivity-oriented designs, simultaneous expression of both antigens increases functional avidity, receptor clustering, or internalization, thereby favoring payload delivery to dual-positive tumor cells over single-positive normal cells. Such designs may produce “AND-gate-like” selectivity, although this terminology should be reserved for molecules in which preferential activity against dual-positive cells has been experimentally demonstrated. In practice, most bsADCs exist along a continuum between these two strategies, and their behavior depends on target density, relative binding affinity, valency, membrane organization, and antibody geometry [10,11,12]. These contrasting OR-gate-like and AND-gate-like design logics are illustrated schematically in Figure 1.

An additional rationale for bispecificity is the functional coupling of antigens with complementary properties. A highly expressed but slowly internalizing antigen may provide broad tumor recognition, whereas a second, rapidly internalizing antigen can facilitate efficient lysosomal delivery of the entire complex. Alternatively, two receptors involved in parallel or compensatory signaling pathways may be targeted to combine payload delivery with pathway inhibition. Several preclinical bsADCs have demonstrated enhanced internalization, broader activity across heterogeneous tumor models, or improved discrimination between dual-positive tumor cells and single-positive normal-tissue surrogates [10,13]. However, these advantages are highly context-dependent and cannot be inferred solely from target co-expression or binding affinity. They must be demonstrated using models that reflect clinically relevant target density, spatial heterogeneity, receptor trafficking, and normal-tissue expression.

Biparatopic ADCs illustrate both the potential and the limitations of enhanced multivalent engagement. Depending on epitope geometry, receptor density, and membrane mobility, binding to two epitopes can support intramolecular engagement, intermolecular receptor crosslinking, higher-order receptor assemblies, and altered intracellular trafficking rather than a single uniform “clustering” mechanism. HER2-directed biparatopic ADCs have shown enhanced internalization and potent activity in models with heterogeneous or relatively low HER2 expression [13]. Nevertheless, the clinical experience with MEDI4276 demonstrated that superior preclinical uptake and cytotoxicity do not necessarily translate into a favorable therapeutic index. Its tetravalent biparatopic architecture was combined with a site-specifically conjugated, highly potent tubulysin payload at an average DAR of approximately 4; therefore, the observed hepatic and gastrointestinal toxicities should be interpreted as an integrated consequence of antibody architecture, target engagement, payload pharmacology, conjugation, and systemic exposure rather than enhanced delivery alone [14,15].

Izalontamab brengitecan, formerly BL-B01D1, currently provides the most clinically advanced and informative example of a dual-antigen bsADC. This EGFR×HER3-directed ADC was designed to exploit the broad and overlapping expression of two ErbB-family receptors across epithelial malignancies while delivering a topoisomerase I inhibitor payload. In a first-in-human phase 1 study, izalontamab brengitecan demonstrated antitumor activity across several heavily pretreated solid tumors and established clinically feasible dosing schedules, although hematologic toxicities were prominent [16]. Subsequent disease-specific studies, including randomized phase 3 evaluation in recurrent or metastatic nasopharyngeal carcinoma, have extended its development beyond exploratory proof of concept [17]. These findings support the clinical feasibility of dual-antigen ADC development but should not be interpreted as universal validation of the bsADC class. The observed activity may reflect a particularly favorable combination of target biology, antibody architecture, payload, dosing strategy, and indication selection.

Indeed, most bsADC programs remain in preclinical or early clinical development, and relatively few have generated sufficient evidence to determine whether bispecificity itself provides a clinically meaningful advantage over optimized monospecific ADCs. Reported programs vary widely in antibody format, valency, target pair, payload class, drug-to-antibody ratio, conjugation method, and experimental comparator. As a result, cross-program comparisons remain difficult. Demonstrating greater activity than an unconjugated antibody or a single parental arm is not sufficient to establish the translational value of a bsADC. A convincing development package should demonstrate that the bispecific architecture confers a reproducible advantage over relevant monospecific ADCs or their combination, remains effective across clinically representative patterns of antigen expression, and preserves adequate pharmacokinetic stability, manufacturability, and safety margins.

The development challenge is further intensified by the convergence of two inherently complex therapeutic platforms. Bispecific antibodies can introduce chain-pairing variants, molecular asymmetry, aggregation, altered Fc interactions, and reduced manufacturing yield. ADC conjugation adds heterogeneity in drug loading, conjugation-site occupancy, linker stability, deconjugation, and free-payload exposure. Bioanalytical assessment may therefore require simultaneous measurement of total antibody, intact conjugate, drug-loaded species, and unconjugated payload. Functional assays must also determine whether binding to each target, simultaneous engagement, internalization, and cytotoxic activity are maintained after conjugation. Consequently, the candidate with the greatest in vitro potency may not be the molecule with the most favorable pharmacokinetics, safety profile, analytical control, or scalability [18,19].

Recent reviews have comprehensively summarized the molecular formats, target combinations, antibody-engineering platforms, linker–payload systems, and emerging pipelines of bsADCs [9,20]. However, less attention has been given to the evidence required to translate a mechanistically attractive bsADC into a clinically developable therapeutic. This distinction is increasingly important as the number of reported candidates expands more rapidly than the number of programs generating interpretable clinical data. Pipeline growth alone does not validate the modality. Clinical success will depend on whether dual targeting produces a measurable advantage that persists across heterogeneous tumors, relevant exposure conditions, scalable manufacturing processes, and biomarker-defined patient populations.

Accordingly, this review evaluates bsADCs through a translational rather than purely structural framework. We first define the principal dual-antigen and biparatopic design strategies and examine the biological rationale for coverage-oriented and co-expression-dependent targeting. We then assess the emerging clinical landscape, using izalontamab brengitecan as an anchor case while critically examining earlier-stage programs according to the maturity of their supporting evidence. Next, we identify the biological, pharmacological, analytical, and manufacturing bottlenecks revealed by current development experience. These observations are subsequently integrated into a practical translational evidence framework organized around target-pair validity, functional proof, translational pharmacology, safety, developability, and biomarker readiness. By focusing on the conditions under which bispecificity generates a clinically meaningful advantage, this review aims to distinguish bsADC designs with genuine translational potential from those that are merely structurally more complex.

## 2. Literature Search Strategy

A structured literature search was conducted using PubMed/MEDLINE, Embase, Web of Science, and Scopus from database inception through 29 July 2026. Search terms included combinations of “bispecific antibody–drug conjugate,” “bispecific ADC,” “dual-antigen ADC,” “dual-targeting ADC,” “biparatopic ADC,” “antibody internalization,” “ADC pharmacokinetics,” “ADC translational development,” and “bispecific antibody manufacturability,” together with the names, development codes, target combinations, and sponsors of individual assets. Reference lists of relevant publications were additionally screened, and ClinicalTrials.gov, the World Health Organization International Clinical Trials Registry Platform, major oncology conference proceedings, and publicly accessible sponsor disclosures were reviewed to identify ongoing or recently reported programs. Peer-reviewed studies were prioritized, while conference abstracts, trial registries, sponsor materials, and publicly available patent information were used as supplementary evidence when recent structural, pharmacokinetic, or development details were unavailable or incomplete in peer-reviewed reports. A formal patent-landscape analysis and subscription chemistry-database search were outside the scope of this review.

The core analysis included ADCs recognizing two distinct antigens, while biparatopic ADCs targeting two non-overlapping epitopes of the same antigen were considered as a related comparator modality. Unconjugated bispecific antibodies, combinations of independently administered ADCs, and monospecific dual-payload ADCs were excluded from the primary pipeline analysis. Owing to substantial heterogeneity in molecular formats, target combinations, payloads, experimental models, and clinical populations, the evidence was synthesized narratively rather than through formal meta-analysis. Development stage, clinical-trial status, and publicly available evidence for individual assets were assessed as of 29 July 2026.

## 3. Design Strategies and Translational Rationale of Bispecific ADCs

### 3.1. Defining the Functional Scope of Bispecific ADCs

Bispecific antibody–drug conjugates combine the binding versatility of a bispecific antibody with the pharmacological complexity of an ADC. In this review, the term bispecific ADC primarily refers to a conjugate whose antibody component recognizes two distinct cell-surface antigens. Biparatopic ADCs, which bind two non-overlapping epitopes on the same antigen, are treated as a structurally distinct but mechanistically related category. Dual-antigen and biparatopic formats often address different primary biological problems, but both can use multivalent engagement, avidity, receptor crosslinking, altered receptor organization, and trafficking to modify payload delivery. They are therefore better viewed as overlapping positions within a continuum of multivalent ADC design rather than as completely separate mechanistic classes.

Bispecificity should not be treated as an independent predictor of therapeutic performance. The biological behavior of a bsADC emerges from the interaction of target density, cellular co-expression, epitope location, arm affinity, valency, molecular geometry, Fc configuration, linker stability, payload properties, and drug-to-antibody ratio. Changes in one design variable may affect several aspects of pharmacology simultaneously. Increasing avidity, for example, may improve retention on dual-positive tumor cells but may also increase normal-tissue uptake or target-mediated clearance when either antigen is broadly distributed. Similarly, accelerated internalization may increase intracellular payload delivery without improving the therapeutic index if the relevant receptor is also accessible in normal tissues. The value of a bispecific architecture must therefore be judged according to the functional advantage it provides over an optimized monospecific ADC, rather than according to structural complexity alone [9,21,22].

The principal design strategies can be organized according to their intended therapeutic function: expanding coverage across heterogeneous tumor-cell populations, increasing selectivity for cells co-expressing two antigens, redirecting or accelerating intracellular trafficking, and combining payload delivery with an additional biological mechanism. These categories are not mutually exclusive. A single molecule may exhibit more than one behavior, but identifying its dominant design logic is essential for selecting appropriate comparators, biomarkers, and translational endpoints.

Molecular architecture provides an additional layer of classification. Dual-binding ADCs may use monovalent 1+1, asymmetric 2+1, symmetric or appended 2+2, and other valency arrangements. These formats can differ substantially in functional avidity, probability of cis-engagement, receptor crosslinking, tissue retention, and target-mediated drug disposition. Epitope geometry further determines whether simultaneous engagement produces an intramolecular complex, intermolecular crosslinking, or higher-order lattice-like receptor organization. Consequently, valency and geometry should be reported together with target pair, linker–payload system, and DAR when comparing bsADC programs [21,22,23].

### 3.2. Coverage-Oriented Dual-Antigen Targeting

A major rationale for dual-antigen targeting is to expand payload delivery across tumors with heterogeneous antigen expression. Conventional monospecific ADCs are most effective when their target is abundant, stable, and uniformly distributed. In solid tumors, however, target expression frequently varies among patients, between primary and metastatic lesions, and among cells within an individual lesion. Antigen-low or antigen-negative subpopulations may consequently receive inadequate payload exposure and survive therapy. A bsADC capable of independently engaging either of two antigens may reduce this form of escape by targeting a broader spectrum of malignant cells. This behavior is often described as “OR-gate-like,” although it represents a pharmacological analogy rather than a strict molecular logic gate.

Coverage-oriented bsADCs require both arms to retain sufficient independent binding and internalization activity. In principle, such a molecule should engage target A-positive/target B-negative, target A-negative/target B-positive, and dual-positive tumor cells. The second specificity may be particularly valuable when paired with a clinically established target that is heterogeneously or weakly expressed. A SORT1×HER2 bsADC, for example, was developed to improve drug delivery in HER2-low tumors by combining HER2 recognition with SORT1, a co-expressed receptor characterized by rapid internalization. The resulting construct showed enhanced cellular binding, internalization, HER2 degradation, cytotoxicity, and in vivo activity in HER2-low models [24]. Because SORT1 contributes both additional binding coverage and rapid trafficking, this example is not a pure OR-gate construct and illustrates the overlap between coverage-oriented and trafficking-assisted design categories.

Broader target recognition, however, does not automatically translate into a wider therapeutic index. Each additional antigen creates an independent route for normal-tissue binding, internalization, and target-mediated drug disposition (TMDD). If either arm retains very high monovalent affinity, abundant single-positive normal tissues or soluble antigen can act as systemic sinks before the conjugate reaches the tumor. Asymmetric affinity tuning is therefore one potential strategy: affinity of one arm can be attenuated so that stable tumor binding depends more strongly on dual-target avidity while binding to single-positive normal tissue is reduced. The useful affinity range is target- and format-specific rather than universal, because antigen density, receptor turnover, epitope accessibility, valency, and internalization jointly determine tissue retention and clearance [21,25].

The incremental value of dual targeting also depends on the linker–payload system. ADCs carrying membrane-permeable payloads can produce bystander killing after intracellular drug release, thereby extending cytotoxicity to adjacent antigen-low or antigen-negative cells. Preclinical studies have shown that locally released, membrane-permeable payloads such as MMAE or DXd can generate greater bystander activity than less permeable payloads [19,26]. Thus, a potent bystander effect may partially compensate for heterogeneous target expression and reduce the additional benefit provided by a second antigen. Conversely, dual targeting may increase the number and spatial distribution of payload-releasing cells within a lesion and thereby amplify local bystander killing. The effects of antibody-mediated coverage and payload diffusion should therefore be distinguished conceptually when interpreting heterogeneous tumor models.

### 3.3. Co-Expression-Dependent Tumor Selectivity

A second strategy uses the coordinated expression of two antigens to improve discrimination between malignant and normal cells. In such constructs, the individual binding arms may be engineered to have relatively modest monovalent affinity, whereas simultaneous engagement of both targets produces stronger overall binding through cross-arm avidity. Productive retention, internalization, or payload delivery is consequently favored on dual-positive cells over cells expressing only one target. This approach is often described as “AND-gate-like,” but that terminology is appropriate only when preferential activity against dual-positive cells has been directly demonstrated.

The EpCAM×CLDN3 bsADC illustrates this strategy. EpCAM is widely expressed in epithelial malignancies but is also present in normal gastrointestinal tissue, limiting the safety of highly potent EpCAM-directed agents. By attenuating the affinities of the individual arms and recovering functional binding through simultaneous engagement of EpCAM and CLDN3, the construct demonstrated preferential binding, internalization, and antitumor activity in dual-positive models while reducing activity against EpCAM-high/CLDN3-low cells used to model normal-tissue risk [11]. For junction-associated targets such as CLDN3, however, measured expression should not be equated directly with antibody accessibility: epithelial polarity, subcellular localization, and disruption of tissue architecture in malignancy can alter the fraction of antigen accessible to a circulating ADC.

A similar avidity-driven approach was used for a P-cadherin (pCAD)×CDH17 bsADC. Lead selection prioritized molecules that bound efficiently to cells co-expressing both antigens but showed limited activity against cells expressing either antigen alone. The resulting MMAE-conjugated molecule demonstrated antitumor activity in dual-positive tumors but not in single-positive comparator tumors [12]. Because MMAE is membrane permeable, however, downstream bystander killing can extend beyond the initially engaged dual-positive cell. Thus, AND-gate-like selectivity should be assessed separately at the levels of antibody binding, internalization, intracellular payload release, and tissue-level cytotoxicity [12,26].

These examples highlight an important distinction between tumor-level prevalence, same-cell co-expression, and physically productive cis-engagement. Independent immunohistochemical positivity for two antigens does not establish that they are present on the same tumor cells, and same-cell optical co-localization does not guarantee that the relevant epitopes are separated by a distance and orientation compatible with simultaneous binding by one bsADC molecule. Receptor density, stoichiometry, membrane mobility, epitope height, and molecular reach can all determine whether dual engagement is sterically feasible. Multiplex immunofluorescence, quantitative digital pathology, spatial proteomics, and single-cell analyses are therefore useful for target-pair mapping, but mechanistic confirmation requires binding and functional assays performed on the final molecular format [21,23,27].

Co-expression must also be sufficiently stable across lesions and treatment states. Strict dependence on two targets may improve tumor selectivity but can reduce coverage if either antigen is absent, heterogeneous, or readily downregulated. This creates an inherent trade-off. Strong AND-gate-like behavior may maximize discrimination between dual-positive tumor cells and single-positive normal tissues, whereas more independent arm activity may increase tumor coverage at the cost of broader tissue binding. Most practical bsADCs are likely to occupy a continuum between these extremes rather than operate as absolute logic gates.

Payload characteristics may further modify this selectivity after internalization. A highly membrane-permeable released drug can diffuse from a dual-positive target cell into surrounding single-positive or antigen-negative cells. This may be advantageous in spatially heterogeneous tumors, but it also means that antibody-level selectivity does not necessarily translate directly into cell-restricted cytotoxicity. The clinically relevant unit of selectivity is therefore the integrated behavior of antibody binding, intracellular trafficking, linker cleavage, payload permeability, and local tissue organization.

### 3.4. Functional Coupling of Target Recognition and Intracellular Trafficking

Some tumor-associated antigens have favorable expression profiles but inefficient ADC trafficking. They may internalize slowly, recycle rapidly, or fail to reach lysosomal compartments that support productive linker cleavage and payload release. Bispecific formats can address this limitation by pairing such an antigen with a second receptor that internalizes rapidly or traffics efficiently to lysosomes. In this guide–effector strategy, one arm provides tumor recognition or broad surface retention, whereas the other alters the intracellular fate of the entire complex.

Experimental work has shown that bispecific internalization is determined by the relative density and trafficking characteristics of both antigens rather than by either arm in isolation. A trafficking-assisted dual-target construct targeting EphA2 and ALCAM demonstrated that the ratio of the two surface antigens could enhance or inhibit internalization depending on cellular context, illustrating that the second target can actively reshape the pharmacology of the first [10]. Similarly, a HER2×CD63 bsADC used the lysosome-associated protein CD63 to redirect HER2-bound complexes toward lysosomal processing and enhance payload delivery [28]. The term “trafficking-assisted” is used here instead of “guide–effector” to avoid confusion with Fc- or immune-effector functions.

This strategy differs from conventional coverage expansion because the second antigen is selected primarily for its intracellular trafficking function rather than for the additional tumor population it identifies. Internalization, recycling to the cell surface, transcytosis, lysosomal degradation, and surface replenishment are distinct processes and should not be treated interchangeably. A highly abundant but poorly internalizing receptor may dominate surface binding and sequester the molecule in a nonproductive state, whereas a lower-density trafficking partner may be sufficient if it efficiently internalizes the ternary complex. Affinity, valency, epitope location, receptor density, and molecular geometry must therefore be optimized together because these parameters determine whether the molecule can engage both targets in cis and whether that engagement changes intracellular routing [21,22,23].

Bispecific design can also combine payload delivery with a second biological mechanism. DB-1419, for example, targets B7-H3 and PD-L1 and was designed to integrate topoisomerase I inhibitor delivery with PD-1/PD-L1 pathway blockade and Fc-mediated immune activity. Preclinical studies reported dual-target binding, intracellular trafficking, direct cytotoxicity, bystander activity, checkpoint inhibition, and immune-mediated effects [13]. Such constructs extend bsADCs beyond dual targeting into multifunctional therapeutics.

The addition of multiple mechanisms can increase biological activity but complicates causal interpretation and dose selection. An observed efficacy advantage may arise from broader tumor recognition, enhanced internalization, signaling inhibition, Fc-mediated activity, payload potency, or interactions among these components. Moreover, the exposure required for receptor or checkpoint occupancy may differ from that required for sufficient intracellular payload delivery; one pharmacological function may therefore plateau while another remains exposure dependent. Multifunctional bsADCs require a clearly defined mechanistic hypothesis and analyte-specific PK/PD plan, and their added complexity is justified only when the contribution of the bispecific architecture can be distinguished from the effects of the payload and unconjugated antibody scaffold.

### 3.5. Biparatopic ADCs as a Mechanistically Related Strategy

Biparatopic ADCs recognize two non-overlapping epitopes on the same antigen. Unlike dual-antigen bsADCs, they do not inherently broaden target coverage across two different proteins. Their principal value is to change the physical organization and intracellular fate of a single receptor system. Depending on antibody format and epitope geometry, biparatopic binding can support intramolecular dual engagement, intermolecular crosslinking, higher-order lattice-like assemblies, altered avidity, and accelerated internalization. These mechanisms overlap with those used by trafficking-assisted dual-antigen bsADCs, supporting their use here as mechanistically related comparators rather than as an entirely separate pharmacological class.

A HER2-directed biparatopic ADC targeting distinct extracellular domains induced receptor reorganization, internalization, lysosomal degradation, and potent activity in models with heterogeneous HER2 expression and resistance to trastuzumab emtansine [13]. Importantly, “receptor clustering” should be understood as a shorthand rather than a single structural mechanism: epitope geometry can favor intermolecular crosslinking and higher-order receptor assemblies, whereas other arrangements may support predominantly intramolecular engagement. These physical differences can substantially influence ADC processing even when the nominal target is unchanged.

The subsequent clinical experience with MEDI4276 provided an important counterpoint. MEDI4276 is a tetravalent biparatopic HER2 ADC targeting extracellular domains 2 and 4, with site-specific conjugation of the tubulysin derivative AZ13599185 through a cleavable linker at an average DAR of approximately 4 [14,15]. Despite strong preclinical uptake and cytotoxicity, dose escalation was limited by gastrointestinal and hepatic toxicities, resulting in a narrow therapeutic window [14]. Clinical bioanalysis separately quantified total antibody, intact or conjugated drug species, deacetylated conjugate species, and unconjugated tubulysin-related analytes [15]. These data do not support attributing toxicity to biparatopic architecture or rapid internalization alone; rather, the outcome reflects the integrated effects of high-affinity HER2 engagement, molecular geometry, payload potency, conjugation chemistry, exposure, and tissue biology.

Biparatopic ADCs are therefore useful comparators for understanding the broader bsADC field. They demonstrate that multivalent receptor engagement and epitope geometry can generate pharmacological behavior not achieved with a conventional monospecific antibody, but they also show that increased cellular delivery must be evaluated together with linker–payload chemistry, DAR, systemic exposure, normal-tissue biology, and therapeutic index. The comparison is mechanistic rather than taxonomic: dual-antigen and biparatopic ADCs remain structurally distinct while sharing several determinants of avidity, trafficking, and disposition.

### 3.6. Integrating Target Biology and Molecular Engineering

The design categories described above are overlapping rather than mutually exclusive. A single bsADC may broaden coverage, favor dual-positive cells, improve internalization, inhibit signaling, and retain Fc-mediated activity. The dominant therapeutic behavior is determined by the interaction between target biology and molecular format. Target-pair selection and format selection should therefore be treated as an iterative co-optimization process: antigen density, epitope geometry, internalization, and normal-tissue distribution determine which valency and architecture are viable, while the chosen architecture can in turn alter apparent target-pair pharmacology.

Several variables are central to this process. Relative arm affinity influences whether the molecule binds independently to either target or depends on dual engagement. Valency and geometry determine accessibility, cooperativity, cis-engagement, receptor crosslinking, and the likelihood of target-mediated sequestration. Antigen density, turnover, soluble antigen, and normal-tissue distribution influence avidity, receptor saturation, tissue penetration, and clearance. The linker–payload system determines whether improved uptake produces localized intracellular killing or a broader bystander effect. Fc configuration may add immune effector activity, but Fcγ-receptor interactions may also alter biodistribution and promote unintended uptake; Fc activity should therefore be considered beneficial or undesirable according to the intended mechanism rather than assumed to be uniformly advantageous.

Accordingly, the key translational question is not whether a candidate binds two antigens, but whether dual recognition produces a reproducible and clinically relevant advantage over an appropriate monospecific alternative. For a coverage-oriented molecule, that advantage may be activity across heterogeneous antigen-expression states. For a co-expression-dependent construct, it may be improved separation between dual-positive tumor cells and single-positive normal tissues. For a trafficking-assisted molecule, it may be more efficient lysosomal delivery and payload release. For a multifunctional construct, it may be the coordinated contribution of payload delivery and a second biological mechanism.

These intended advantages define the evidence that should be generated before clinical development. However, mechanistic plausibility alone is insufficient. The benefit of bispecificity must remain detectable after conjugation and must be compatible with acceptable pharmacokinetics, safety, manufacturability, and biomarker feasibility. These considerations support a principle of functional design parsimony: the least complex ADC architecture capable of achieving the predefined biological and translational objective should be preferred, and added bispecific or multifunctional complexity should be justified by measurable incremental value over a simpler alternative. The principal design categories, representative examples, intended advantages, and key development risks are summarized in Table 1, while Figure 1 provides a schematic overview of their functional mechanisms. The preclinical evidence needed to determine whether these design concepts are sufficiently robust for clinical translation is discussed in the following section.

## 4. Clinical Development Landscape and Emerging Evidence

As of 29 July 2026, the clinical development of bispecific antibody–drug conjugates remains uneven. Izalontamab brengitecan has progressed from first-in-human evaluation to disease-specific expansion and randomized phase III studies, providing the most mature evidence for a dual-antigen bsADC. JSKN016 has generated preliminary phase I activity in HER2-negative breast cancer and has entered randomized phase III evaluation, although its clinical results remain available only in conference-abstract form. Most other dual-antigen programs remain in dose escalation or early expansion without publicly reported outcome data. In parallel, HER2-directed biparatopic ADCs have produced contrasting clinical experiences, ranging from the restricted therapeutic index of MEDI4276 to the more favorable early profile of TQB2102. The available evidence therefore establishes the clinical feasibility of dual-binding ADCs but does not yet demonstrate that bispecificity is uniformly superior to optimized monospecific targeting.

### 4.1. Izalontamab Brengitecan as the Principal Clinical Anchor

Izalontamab brengitecan, formerly BL-B01D1, is an EGFR×HER3 bsADC built on an appended 2+2 architecture in which an anti-EGFR IgG is combined with two anti-HER3 single-chain variable fragments. The antibody is conjugated through a cathepsin-cleavable linker to Ed-04, a camptothecin-derived topoisomerase I inhibitor, at a reported average DAR of approximately 8 [16,32]. This architecture differs materially from earlier biparatopic microtubule-inhibitor ADCs in valency, target biology, payload class, and drug loading and therefore should not be interpreted as a simple test of bispecificity alone.

In the first-in-human phase I study, 195 patients with heavily pretreated locally advanced or metastatic solid tumors received izalontamab brengitecan using weekly, split-dose, or once-every-three-week schedules. Four dose-limiting events of febrile neutropenia occurred during dose escalation. Among 174 efficacy-evaluable patients, the objective response rate was 34%. Treatment-related adverse events were predominantly hematologic, and grade ≥ 3 events occurred in 71% of patients. Based on the combined safety and activity findings, 2.5 mg/kg administered on days 1 and 8 of each 21-day cycle was selected as the recommended phase II dose [16].

The study established several principles that subsequently shaped the program. First, a high-DAR topoisomerase I inhibitor bsADC could be delivered at clinically relevant exposure using a split-dose regimen. Second, activity across multiple tumor types supported disease-specific expansion rather than continued development as a nonspecific basket program. Third, the dominant hematologic toxicity suggested that payload class and systemic exposure remained major determinants of tolerability. The rationale for split dosing should therefore be evaluated with analyte-specific metrics such as Cmax, AUC, trough concentration, cumulative conjugated-drug exposure, and free-payload exposure rather than tolerability alone. The study did not determine how much clinical activity was attributable specifically to EGFR×HER3 dual recognition rather than to the potency of the Ed-04 payload, engagement of either individual receptor, or bystander killing.

#### 4.1.1. Disease-Specific Expansion

Subsequent studies demonstrated reproducible activity in defined disease settings. In a phase Ib study involving 82 patients with previously treated metastatic esophageal squamous cell carcinoma, the confirmed objective response rate was 29.3% in the overall population. At the selected 2.5 mg/kg dose, the confirmed response rate was 39.6% and the disease-control rate was 79.2%. Grade ≥ 3 treatment-related adverse events occurred in 63.3% of patients treated at this dose and remained primarily hematologic. Two cases of grade ≥ 3 interstitial lung disease were reported, identifying pulmonary toxicity as an uncommon but clinically relevant risk requiring continued surveillance [33].

In EGFR-mutated NSCLC after progression on EGFR tyrosine kinase inhibitors, a pooled analysis of phase I and II studies included 171 patients. The confirmed objective response rate was 47.4%, median duration of response was 8.5 months, median progression-free survival was 6.9 months, and median overall survival was 24.8 months. Among chemotherapy-naïve patients treated after EGFR-TKI failure at the recommended dose, the response rate was 56.0% and median progression-free survival was 12.5 months. Grade ≥ 3 treatment-related adverse events occurred in 70.2% of patients and were again predominantly hematologic. Because the analysis was pooled and post hoc and did not include a randomized comparator, the findings were appropriately characterized as hypothesis-generating [34].

Activity has also been reported in NSCLC harboring actionable genomic alterations outside classical EGFR mutations. In a phase Ib study of 83 patients with EGFR exon 20 insertions or nonclassical EGFR mutations, HER2 mutations, KRAS/BRAF/MET mutations, or ALK/ROS1/RET/NTRK fusions, the confirmed response rate was 39.7% and median progression-free survival was 7.0 months. Responses were particularly notable in the EGFR exon 20 insertion/nonclassical mutation and HER2-mutant cohorts. These findings further support broad activity across molecularly heterogeneous NSCLC, although the relationship between receptor expression, genomic alterations, and response remains incompletely defined [35].

In previously treated extensive-stage small-cell lung cancer, a phase Ib study enrolled 52 patients and reported an objective response rate of 48.1%, median progression-free survival of 4.1 months, and median overall survival of 12.2 months. Among 22 patients treated in the second-line setting, the response rate was 72.7%, median progression-free survival was 6.2 months, and median overall survival was 15.0 months. Exploratory analyses suggested that HER3-positive tumors might derive greater benefit, but this association requires prospective confirmation and does not yet constitute a validated patient-selection strategy [36].

Together, these disease-specific studies demonstrate that izalontamab brengitecan has reproducible antitumor activity across histologically and molecularly diverse malignancies. They also expose a central biomarker uncertainty. Clinical enrollment has generally not required a validated EGFR/HER3 co-expression threshold, and available studies have not established whether response depends on same-cell co-expression, high expression of either receptor, tumor-wide coverage of complementary cell populations, receptor trafficking, or intrinsic sensitivity to the Ed-04 payload. The observed activity is compatible with a coverage-oriented dual-targeting mechanism, but direct clinical demonstration that bispecificity expands the responder population remains limited.

#### 4.1.2. Randomized Phase III Evidence

The transition from single-arm expansion to randomized evaluation represents the most important development in the bsADC field. In the phase III BL-B01D1-303 study, 386 patients with heavily pretreated recurrent or metastatic nasopharyngeal carcinoma were randomized to izalontamab brengitecan or physician’s-choice chemotherapy. At the first planned interim analysis, the objective response rate by masked independent central review was 54.6% with izalontamab brengitecan and 27.0% with chemotherapy. Overall-survival data were immature at the time of the peer-reviewed publication [17].

This study provides the first peer-reviewed randomized phase III evidence that a dual-antigen bsADC can produce superior response activity relative to standard chemotherapy. Its interpretation nevertheless requires precision. The published interim analysis established an improvement in response rate, but it did not yet establish a mature survival benefit. Moreover, substantial hematologic toxicity remained, indicating that improved antitumor activity was not achieved by eliminating payload-associated systemic toxicity. The study validates izalontamab brengitecan as a clinically active drug and supports the EGFR×HER3/Ed-04 configuration, but it does not independently quantify the contribution of bispecificity.

Additional randomized phase III findings were reported at the 2026 ASCO Annual Meeting. In recurrent or metastatic esophageal squamous cell carcinoma after progression following first-line PD-1/PD-L1 inhibitor plus platinum-based chemotherapy, izalontamab brengitecan improved median overall survival to 9.8 months (95% CI, 7.9–11.0) compared with 7.2 months (95% CI, 6.2–8.2) with chemotherapy (HR, 0.64; 95% CI, 0.49–0.83). Median progression-free survival was 4.2 months (95% CI, 3.6–4.5) versus 2.0 months (95% CI, 1.6–2.7), respectively (HR, 0.50; 95% CI, 0.40–0.63). Both prespecified primary endpoints were met at the interim analysis. These results remained available in conference-abstract form as of the review cutoff [37].

In a separate randomized phase III trial involving 418 patients with previously treated unresectable locally advanced or metastatic triple-negative breast cancer, median progression-free survival was 8.5 months with izalontamab brengitecan and 3.1 months with physician’s-choice chemotherapy (HR, 0.29; 95% CI, 0.22–0.38). Median overall survival was 15.9 and 12.5 months, respectively (HR, 0.60; 95% CI, 0.42–0.85), and confirmed response rates were 51.7% and 20.5% (odds ratio, 4.28; 95% CI, 2.75–6.66). The trial met both dual-primary endpoints at the prespecified interim analysis. These findings were reported in conference-abstract form and await full peer-reviewed publication [38].

The current randomized evidence should therefore be described according to publication maturity. Peer-reviewed phase III evidence has established superior response activity in nasopharyngeal carcinoma, whereas conference-reported phase III studies in esophageal squamous cell carcinoma and triple-negative breast cancer have additionally reported progression-free and overall-survival benefits. Collectively, these results move izalontamab brengitecan beyond exploratory proof of concept. However, they are best interpreted as molecule-level and target-pair-level validation rather than universal validation of the bsADC class.

### 4.2. JSKN016 as an Emerging Late-Stage Dual-Antigen bsADC

JSKN016 is a TROP2×HER3 bsADC site-specifically conjugated through glycan engineering to a topoisomerase I inhibitor via a cleavable linker, producing a reported DAR of 4 [39]. This controlled conjugation strategy is intended to reduce product heterogeneity relative to less controlled loading approaches. Mechanistically, the TROP2×HER3 pair should not be assumed to function only as a coverage strategy: depending on antigen density, epitope geometry, and arm affinity, productive dual engagement may also influence avidity or internalization. Current clinical data do not yet establish which of these mechanisms predominates.

At the 2026 ASCO Annual Meeting, phase I results were reported in HER2-negative locally advanced or metastatic breast cancer. Among 31 patients with triple-negative breast cancer, the independent-review committee-assessed unconfirmed response rate was 61.3% and median progression-free survival was 7.9 months. Among 29 patients with hormone-receptor-positive/HER2-negative breast cancer, the corresponding response rate was 55.2% and median progression-free survival was 11.1 months. These findings provide a meaningful early clinical signal, but they remain based on a meeting abstract, relatively small cohorts, and a nonrandomized study [39].

JSKN016 has nevertheless advanced rapidly into randomized development. A phase III study comparing JSKN016 with physician’s-choice therapy in patients with unresectable locally advanced, recurrent, or metastatic triple-negative breast cancer after at least two prior lines of systemic therapy began in March 2026. The coprimary endpoints are progression-free survival by blinded independent review and overall survival, with an estimated enrollment of 364 patients (NCT07533123).

The progression of JSKN016 illustrates that the bsADC landscape is no longer represented solely by izalontamab brengitecan and a collection of dose-escalation programs. However, JSKN016 should not yet be considered clinically validated. Its phase I signal requires confirmation in a randomized setting, and correlative analyses will be needed to establish whether activity is associated with TROP2 expression, HER3 expression, same-cell co-expression, complementary tumor-cell coverage, or general sensitivity to the topoisomerase I inhibitor payload.

### 4.3. Clinical Lessons from Biparatopic ADCs

Clinical experience with HER2-directed biparatopic ADCs provides a mechanistically relevant comparison. These molecules bind two non-overlapping epitopes on HER2 and are designed primarily to enhance avidity, receptor clustering, internalization, and lysosomal trafficking rather than to expand coverage across two distinct antigens.

MEDI4276 was a tetravalent biparatopic HER2 ADC targeting extracellular domains 2 and 4 and carrying the highly potent tubulysin derivative AZ13599185 through site-specific conjugation, with an average DAR of approximately 4 [14,15]. In a first-in-human dose-escalation study involving 47 heavily pretreated patients with HER2-positive breast or gastric cancer, one complete response and two partial responses were reported. Dose-limiting elevations in hepatic enzymes occurred at multiple dose levels, the maximum tolerated dose was exceeded at 0.9 mg/kg, and doses above 0.3 mg/kg were considered poorly tolerated [14]. The outcome should therefore be interpreted as a property of the complete tetravalent HER2–linker–tubulysin configuration rather than as evidence that biparatopic targeting itself is intrinsically toxic.

The MEDI4276 program also demonstrated the analytical complexity that may accompany advanced ADC architectures. Clinical bioanalysis required separate characterization of total antibody, conjugated payload species, deacetylated conjugate species, and unconjugated tubulysin-related analytes to interpret exposure, metabolism, and toxicity [15].

A more favorable clinical profile has been reported with TQB2102, an asymmetric biparatopic ADC recognizing HER2 extracellular domains 2 and 4. TQB2102 uses an enzyme-cleavable GGFG-type linker and a deuterated DXd-related topoisomerase I inhibitor payload at a reported DAR of approximately 5.8–6.0 [31]. In a phase I study of 195 patients with advanced solid tumors, no dose-limiting toxicities were observed and the maximum tolerated dose was not reached. Doses of 6.0 and 7.5 mg/kg every three weeks were selected for further development. Objective response rates were 52.4% in HER2-positive metastatic breast cancer, 38.7% in HER2-positive colorectal cancer, 40.0% in HER2-positive gastric or gastroesophageal-junction cancer, and 47.2% in HER2-low metastatic breast cancer. Grade ≥ 3 toxicities were primarily hematologic, and drug-related interstitial lung disease occurred in one patient [31].

The contrasting experiences of MEDI4276 and TQB2102 indicate that biparatopic targeting is neither inherently safe nor inherently toxic. Both engage HER2 extracellular domains 2 and 4, but MEDI4276 combines a tetravalent high-affinity HER2 architecture with a site-specific tubulysin payload at DAR ~4, whereas TQB2102 uses an asymmetric biparatopic format with a cleavable linker and a topoisomerase I payload at DAR ~6 [14,15,31]. Their different clinical profiles therefore cannot be attributed to epitope pair alone and likely reflect the integrated effects of valency, affinity, linker chemistry, payload class and potency, DAR, conjugation strategy, systemic stability, dose, receptor trafficking, and tumor biology. This comparison is directly relevant to dual-antigen bsADCs: bispecificity cannot be evaluated independently of the complete molecular configuration.

The principal clinical-stage dual-antigen and biparatopic ADC programs are summarized in Table 2, together with their target design, payload class, and current translational interpretation.

### 4.4. Expansion of the Early Clinical Pipeline

Beyond izalontamab brengitecan and JSKN016, multiple dual-antigen bsADCs have entered first-in-human evaluation. These programs test a range of biological hypotheses, including broader coverage of heterogeneous tumor populations, co-expression-dependent avidity, and functional coupling of complementary targets. However, trial initiation should not be interpreted as evidence of clinical activity.

IDE034 is being evaluated in a phase Ia/Ib study involving selected solid tumors expressing B7-H3 and PTK7. The study includes dose escalation and biomarker-oriented expansion and represents a direct clinical test of a target pair intended to identify overlapping tumor populations while delivering a topoisomerase I inhibitor payload (NCT07503808).

NEOK001 is a B7-H3×ROR1 exatecan-based bsADC undergoing phase I evaluation in selected solid tumors, whereas NEOK002 targets EGFR and MUC1 in a similarly structured first-in-human study. As no clinical outcome data were publicly available by 29 July 2026, these programs should be described according to their target pairs, molecular designs, biomarker strategies, and trial status without assigning presumed efficacy (NCT07612176; NCT07612189).

For clarity and durability, detailed information on these and other early assets is best presented in a pipeline table containing the target pair, antibody format, linker–payload system, indication, clinical phase, trial identifier, biomarker criteria, and highest level of publicly available evidence. The narrative text should remain focused on clinically interpretable results rather than reproducing a rapidly changing list of trial initiations.

### 4.5. Cross-Program Clinical Insights

Several early conclusions can be drawn from the available clinical experience.

First, late-stage clinical feasibility has been demonstrated, but only for a limited number of molecular designs. Izalontamab brengitecan has generated randomized phase III evidence across several diseases, while JSKN016 has progressed to phase III on the basis of preliminary phase I findings. TQB2102 further indicates that a biparatopic ADC can achieve substantial systemic dosing and activity across HER2-positive and HER2-low tumors.

Second, clinical activity does not by itself prove the value of bispecificity. No randomized study has directly compared a dual-antigen bsADC with a linker-, payload-, DAR-, and dose-matched monospecific ADC. Superiority over chemotherapy validates the complete drug product but does not isolate the contribution of the second binding arm. Cross-trial comparisons with established monospecific topoisomerase I ADCs can provide clinical context, but differences in indication, prior therapy, target expression, dose, and payload make such comparisons unsuitable for mechanistic attribution. Biomarker, PK, tissue-distribution, and mechanistic analyses remain necessary to determine whether bispecificity broadens tumor coverage, improves internalization, or enhances selectivity in patients.

Third, toxicity remains strongly influenced by the linker–payload system and systemic exposure. Hematologic toxicity is prominent with izalontamab brengitecan, JSKN016, and TQB2102, all of which use topoisomerase I inhibitor payloads. MEDI4276 produced a different hepatic and gastrointestinal pattern with a tubulysin-based payload. The clinical safety profile of a bsADC therefore cannot be attributed to target biology alone.

Fourth, dose and schedule form part of the molecular strategy. The split-dose regimen selected for izalontamab brengitecan illustrates that schedule optimization may improve clinical deliverability when peak exposure or cumulative payload toxicity limits conventional once-per-cycle dosing. Dose selection should integrate analyte-specific AUC, Cmax, trough concentration, exposure–toxicity, exposure–response, target saturation, recovery of vulnerable tissues, dose intensity, and durability of tumor payload delivery rather than relying on nominal mg/kg dose alone.

Fifth, biomarker development remains less mature than drug development. Most studies have not prospectively validated a dual-expression algorithm. Clinical trials frequently enroll broad disease populations and assess EGFR, HER3, TROP2, or other targets retrospectively or exploratorily. Future studies should distinguish whether response is associated with expression of either target, same-cell co-expression, spatial complementarity, receptor internalization, or a continuous composite score.

Finally, the field should be judged according to evidence maturity rather than pipeline size. Peer-reviewed randomized evidence, reproducible disease-specific activity, validated dose–exposure relationships, interpretable biomarker associations, and manageable long-term toxicity provide stronger validation than the number of assets entering phase I. On this basis, izalontamab brengitecan currently remains the principal clinical anchor; JSKN016 represents an emerging late-stage dual-antigen program; TQB2102 provides encouraging biparatopic evidence; MEDI4276 remains an important cautionary example; and most other programs should still be considered clinically testable hypotheses.

## 5. Translational Bottlenecks and Development Priorities

The increasing number of bispecific antibody–drug conjugates entering development demonstrates the technical feasibility and growing interest in this modality. However, pipeline expansion should not be equated with clinical maturity. Most dual-antigen bsADCs remain in preclinical or early clinical development, and even the most advanced programs have not established that bispecificity consistently provides superior efficacy or a wider therapeutic index than an optimized monospecific ADC. The central challenge is therefore no longer whether an ADC can be engineered to recognize two targets, but whether the second specificity creates sufficient biological and clinical value to justify the additional pharmacological, analytical, manufacturing, and regulatory complexity.

The major translational priorities can be organized around five questions: whether the target pair provides a nonredundant biological advantage; whether the molecular design improves therapeutic index rather than cellular potency alone; whether the contribution of bispecificity can be distinguished from that of the linker–payload system; whether the molecule can be manufactured and controlled reproducibly; and whether early clinical development can generate interpretable biomarker, exposure–response, and dose-optimization evidence.

### 5.1. Moving from Individual Target Prevalence to Target-Pair Biology

A major limitation in current target selection is the reliance on the independent prevalence of two antigens. Two targets may each be frequently expressed in a cancer type without forming a therapeutically useful pair. The biological requirements differ according to the intended bsADC design. A coverage-oriented molecule should meaningfully expand the fraction or spatial distribution of malignant cells accessible to the ADC. A co-expression-dependent molecule requires both antigens on the same cells at densities and orientations compatible with simultaneous engagement. A trafficking-assisted construct requires the second target to improve internalization or intracellular routing rather than merely provide additional surface binding.

Preclinical EpCAM×CLDN3, P-cadherin×CDH17, and CD33×CD7 programs have demonstrated that reduced monovalent affinity can be recovered through avidity on dual-positive cells, thereby improving discrimination from relevant single-positive populations [11,12,29]. These studies support the concept of a composite tumor antigen. However, the translational value of such a strategy depends on whether the dual-positive population is sufficiently prevalent, spatially accessible, and stable in the intended clinical setting.

Development programs should therefore generate quantitative target-pair maps rather than rely solely on independent expression estimates. Relevant variables include same-cell co-expression, antigen density, spatial proximity, epitope accessibility, receptor stoichiometry, intratumoral heterogeneity, discordance among metastatic lesions, and changes following treatment. Such maps should be treated as discovery evidence rather than direct proof of simultaneous molecular engagement, which also depends on nanometer-scale geometry and receptor mobility. Archival primary-tumor tissue may not adequately represent heavily pretreated metastatic disease, particularly when previous targeted therapies or ADCs have selected for antigen-low subclones.

Biomarker design should reflect the proposed therapeutic mechanism. A coverage-oriented molecule may require an algorithm that measures the proportion of tumor cells expressing either antigen and the spatial complementarity of those populations. A co-expression-dependent construct may require a dual-positive cell fraction or quantitative co-expression score. A trafficking-assisted molecule may require sufficient expression of both the tumor-recognition antigen and the internalizing partner. Separate binary immunohistochemical cutoffs for each target may be operationally convenient but may not adequately represent these biological relationships.

The clinical development of izalontamab brengitecan illustrates this unresolved issue. The molecule has demonstrated reproducible activity and randomized efficacy without a prospectively validated EGFR/HER3 dual-expression algorithm [16,17,33]. These findings validate the complete drug product but do not establish whether clinical benefit is associated with expression of either receptor, same-cell co-expression, complementary coverage of heterogeneous tumor populations, or sensitivity to the topoisomerase I inhibitor payload. Prospective tissue analyses should therefore be incorporated into future trials to distinguish these possibilities.

### 5.2. Improving Therapeutic Index Rather than Maximizing Potency

The second bottleneck is the tendency to prioritize strong binding, rapid internalization, and low in vitro IC_50_ values during lead selection. These properties may increase cellular payload delivery but do not necessarily produce a superior clinical drug. Increased normal-tissue uptake, target-mediated clearance, unstable conjugation, or excessive systemic payload exposure may eliminate an apparent preclinical efficacy advantage.

The contrasting clinical profiles of MEDI4276 and TQB2102 show that enhanced receptor engagement is not an independent determinant of therapeutic index. Both are HER2-directed biparatopic ADCs, but their tolerability differed substantially, indicating that the complete antibody–linker–payload configuration, rather than biparatopic binding alone, determines clinical behavior [14,31].

Toxicity is frequently influenced by payload class, linker stability, DAR, and systemic exposure to unconjugated payload, although target-dependent toxicities may also occur [40]. The hematologic toxicities observed with several topoisomerase I inhibitor-containing bsADCs demonstrate that adding a second target does not remove the intrinsic pharmacology of the cytotoxic component. Bispecificity may alter tissue distribution, but safety cannot be interpreted independently of the linker–payload system.

Lead optimization should therefore focus on the differential delivery advantage rather than on absolute uptake or potency. For a co-expression-dependent molecule, the relevant measure is the separation in binding, internalization, and payload delivery between dual-positive tumor cells and clinically relevant single-positive normal cells. For a coverage-oriented bsADC, the increase in tumor-cell coverage should be weighed against the additional normal-tissue distribution introduced by the second target. For a trafficking-assisted construct, improved lysosomal delivery should be balanced against the expression and internalization of the trafficking receptor in normal tissues.

Affinity optimization may consequently be more valuable than affinity maximization. Attenuated individual-arm affinity can preserve avidity on dual-positive tumor cells while reducing binding to single-positive tissues, but the desired kinetic change may arise from a lower association rate, faster dissociation rate, or both, with different consequences for tissue retention and internalization. There is no validated universal thermodynamic window for bsADC affinity attenuation; the appropriate range depends on antigen density, geometry, valency, receptor kinetics, payload potency, and clinically achievable exposure. Similar trade-offs apply to DAR, linker cleavage, and payload permeability. The optimal candidate may not have the greatest in vitro potency, but rather the widest exposure range over which antitumor activity remains separated from clinically relevant toxicity.

Tumor penetration should also be considered. High-affinity binding to abundant antigens can result in perivascular sequestration and heterogeneous microscopic distribution. This problem may be amplified when either arm of a bsADC binds strongly to an antigen concentrated near tumor vasculature. Experimental and modeling studies indicate that affinity moderation, carrier dosing, or altered dosing schedules may improve intratumoral distribution under selected conditions, although the effect depends on antigen density, payload potency, and the total amount of conjugated drug delivered per cell [41,42]. These strategies should therefore be evaluated within an integrated efficacy and safety framework rather than assumed to be universally beneficial.

### 5.3. Distinguishing Bispecificity from Payload Activity and Resistance Biology

A third challenge is determining whether observed activity is attributable to the bispecific architecture. Many emerging bsADCs use highly active topoisomerase I inhibitor payloads, cleavable linkers, and membrane-permeable released drugs. These characteristics can generate substantial activity and bystander killing even with monospecific targeting. Without appropriately matched comparators, the contribution of the second binding arm may be overstated.

Preclinical comparator studies should therefore match the antibody backbone, linker, payload, conjugation site, DAR, and exposure as closely as technically feasible. Payload-matched monospecific ADCs are particularly important because comparisons with an unconjugated antibody, free payload, or untreated control establish drug activity but do not establish the incremental value of bispecificity. A combination of the corresponding monospecific ADCs may provide additional information, although differences in total antibody and payload exposure must be considered.

Clinical attribution is more difficult. Superiority to chemotherapy validates the complete bsADC product but does not isolate whether efficacy results from dual recognition, one dominant target, bystander killing, or payload sensitivity. Direct randomized comparison with a matched monospecific ADC may not be practical when no equivalent clinical asset exists. The contribution of bispecificity will therefore often need to be supported through convergent evidence from tissue biomarkers, PK, pharmacodynamic assays, target-expression heterogeneity, and patterns of clinical response.

Resistance should also be incorporated into target-pair selection. ADC resistance can arise through loss or downregulation of the surface antigen, altered internalization and lysosomal processing, drug-efflux transporters, changes in the intracellular payload target, or impaired apoptotic response. Analysis of sacituzumab govitecan resistance identified alterations affecting both TROP2 and TOP1, demonstrating that resistance to the antibody-targeting and payload components can evolve independently within the same patient [4].

Dual targeting may reduce escape caused by loss of one antigen, but it cannot necessarily overcome resistance shared by the payload class. This is increasingly relevant because many bsADCs use related topoisomerase I inhibitors. Sequential administration of ADCs carrying similar payloads may therefore produce cross-resistance despite changing the antibody targets. Early clinical studies should document prior ADC exposure, payload class, response duration, and mechanisms of progression.

The anticipated resistance pattern also differs by design. Coverage-oriented bsADCs may retain activity following loss of one target if the remaining arm provides sufficient binding and internalization. Strictly co-expression-dependent molecules may lose avidity when either antigen is reduced. Trafficking-assisted constructs may fail following loss of the internalizing partner even when the primary recognition antigen remains abundant. These potential failure modes should be examined in preclinical escape models and, where feasible, through paired clinical biopsies or circulating-tumor DNA analyses.

### 5.4. Integrating CMC and Analytical Development with Molecular Design

Another major bottleneck is the delayed recognition of developability risk. BsADCs combine the assembly and stability challenges of engineered bispecific antibodies with the heterogeneity introduced by conjugation. A biologically compelling molecule may become unsuitable for development because of chain mispairing, low expression, aggregation, fragmentation, hydrophobicity, poor formulation stability, uncontrolled DAR, or an altered balance between the two binding arms after conjugation.

Studies of unconjugated bispecific antibodies have identified greater risks of aggregation, fragmentation, self-association, and interfacial instability in some engineered formats compared with conventional IgGs [43]. Although these studies were not conducted with conjugated bsADCs, the underlying assembly and stability liabilities may be amplified by the hydrophobic and structural effects of payload conjugation.

Chain pairing is particularly important for asymmetric IgG-like formats. Heavy-chain homodimerization, heavy-chain/light-chain mispairing, and broader chain-scrambling variants are mechanistically distinct defects and may require different engineering solutions and analytical methods. Mispaired species may resemble the intended product and may not be sufficiently resolved by conventional platform methods. Increased levels of mispaired variants have been associated with reduced biological potency, demonstrating that these species can affect function rather than merely analytical appearance [44].

CMC development should therefore begin during lead selection. The intended mechanism should determine which quality attributes are critical. Loss of one binding arm may reduce coverage in an OR-gate-like design, whereas a modest shift in relative arm affinity may disrupt the selectivity of an avidity-dependent construct without substantially changing conventional single-antigen binding results. A trafficking-assisted molecule may require a functional assay sensitive to internalization or intracellular payload delivery rather than binding alone.

The final analytical strategy may need to characterize molecular identity, chain pairing, aggregates, fragments, charge variants, conjugation-site occupancy, DAR distribution, free payload, linker stability, individual-arm binding, simultaneous engagement, and mechanism-relevant potency. Orthogonal chromatographic, electrophoretic, mass-spectrometric, and biophysical methods should be selected according to the critical quality attribute being measured. Not every mechanistic assay needs to become a formal release assay, but the relationship between product variation and functional performance should be understood, and dual-antigen functional assays should be considered when the proposed mechanism depends on coordinated engagement of both targets.

Manufacturing changes involving cell line, production scale, purification, conjugation, or formulation may alter both conventional quality attributes and the functional balance between the two arms. Comparability exercises should therefore assess whether post-change material preserves relevant physicochemical properties, biological activities, and payload-delivery functions. This approach is consistent with ICH Q5E, which applies comparability principles to biotechnology-derived proteins and their conjugates and emphasizes evaluating whether manufacturing changes adversely affect product quality, safety, or efficacy [45].

### 5.5. Generating More Informative Early Clinical Evidence

Early clinical development should move beyond a predominantly maximum-tolerated-dose-oriented approach. ADC efficacy and toxicity may be associated with different circulating analytes and may plateau at different exposure levels. Increasing dose after tumor delivery or target engagement has approached saturation may increase systemic payload exposure without producing a proportional increase in antitumor activity.

The FDA ADC clinical pharmacology guidance recommends a product-specific strategy addressing bioanalytical methods, dosing, dose- and exposure–response relationships, intrinsic factors, immunogenicity, and drug–drug interactions [46]. Depending on the molecular design and linker–payload system, relevant analytes may include total antibody, conjugated ADC, unconjugated payload, and active catabolites. For bsADCs, the interpretation is further complicated because either target may contribute to nonlinear clearance, tissue sequestration, and dose-dependent target saturation.

Dose escalation should characterize a sufficiently broad exposure range to identify where pharmacological activity and major toxicities begin to plateau or diverge. Relevant variables include AUC, Cmax, trough exposure, time above a pharmacologically active concentration, cumulative conjugated-payload exposure, free-payload exposure, dose intensity, and target occupancy where measurable. First-cycle DLTs may not capture cumulative cytopenias, interstitial lung disease, neuropathy, ocular toxicity, or repeated dose modifications. Treatment interruptions, recovery between administrations, discontinuation rates, and patient-reported tolerability should therefore contribute to dose selection.

Schedule should be treated as part of the therapeutic design. The split-dose regimen selected for izalontamab brengitecan illustrates how fractionation may alter peak exposure, recovery of vulnerable normal tissues, and cumulative dose delivery. However, schedule optimization should be supported by analyte-specific PK and exposure–response analyses rather than empirical tolerability alone.

When several biologically active doses remain plausible, randomized dose evaluation may be warranted before initiation of a registrational trial. The FDA’s 2024 oncology dosage guidance, consistent with the principles advanced through Project Optimus, emphasizes identifying a dosage that optimizes benefit–risk during clinical development rather than defaulting to the highest tolerated dose [47]. This principle is particularly relevant to bsADCs because dual-target disposition and multiple pharmacologically active components may produce different exposure plateaus for efficacy and toxicity.

Model-informed drug development can support this process by integrating systemic exposure, antigen density, receptor turnover, internalization, tumor penetration, payload release, and response. Translation of ADC dosing from xenograft models to humans remains challenging, and apparent preclinical efficacy should be interpreted in the context of clinically achievable antibody-equivalent doses and tumor payload concentrations [48]. Models should be used to identify sensitive assumptions and guide data collection, not to replace empirical comparison of clinically plausible doses and schedules.

Early clinical trials should also generate evidence capable of testing the hypothesized value of bispecificity. Prespecified tissue analyses, serial circulating biomarkers, multiple PK analytes, pharmacodynamic measurements, and biopsies at progression may help determine whether activity is associated with target A, target B, dual expression, spatial complementarity, enhanced internalization, or payload sensitivity. Without such evidence, even a successful clinical trial may validate the drug without validating its proposed bispecific mechanism.

### 5.6. Rationale for an Integrated Translational Framework

Taken together, current clinical experience and the translational bottlenecks described above indicate that bsADC development requires a structured evidence framework that links target-pair biology, molecular design, pharmacology, safety, developability, and biomarker readiness. The purpose of such a framework is not to maximize molecular complexity, but to identify early whether the second specificity provides sufficient incremental value to justify additional pharmacological, analytical, manufacturing, and regulatory burden.

More complex approaches, including conditionally activated constructs, multispecific ADCs, and dual-payload conjugates, may eventually expand the modality. However, each additional function creates new dependencies, quality attributes, analytical requirements, and potential failure modes. Such designs should be pursued when they address a defined biological limitation rather than because molecular complexity is presumed to increase efficacy.

We therefore propose functional design parsimony as a guiding principle for bsADC development: the use of the least complex molecular architecture capable of achieving the required tumor coverage, cellular selectivity, intracellular delivery, and therapeutic index. Operationally, additional complexity should be justified against a simpler alternative across efficacy, selectivity, pharmacokinetics, toxicity, biomarker feasibility, analytical control, and manufacturability. BsADCs are most likely to succeed when dual targeting is treated not as the intrinsic value of the product, but as one engineering solution to a precisely characterized translational problem. The following section operationalizes these principles as domain-specific evidence requirements and decision gates.

## 6. Proposed Translational Evidence Framework for bsADC Development

**Figure 2:**
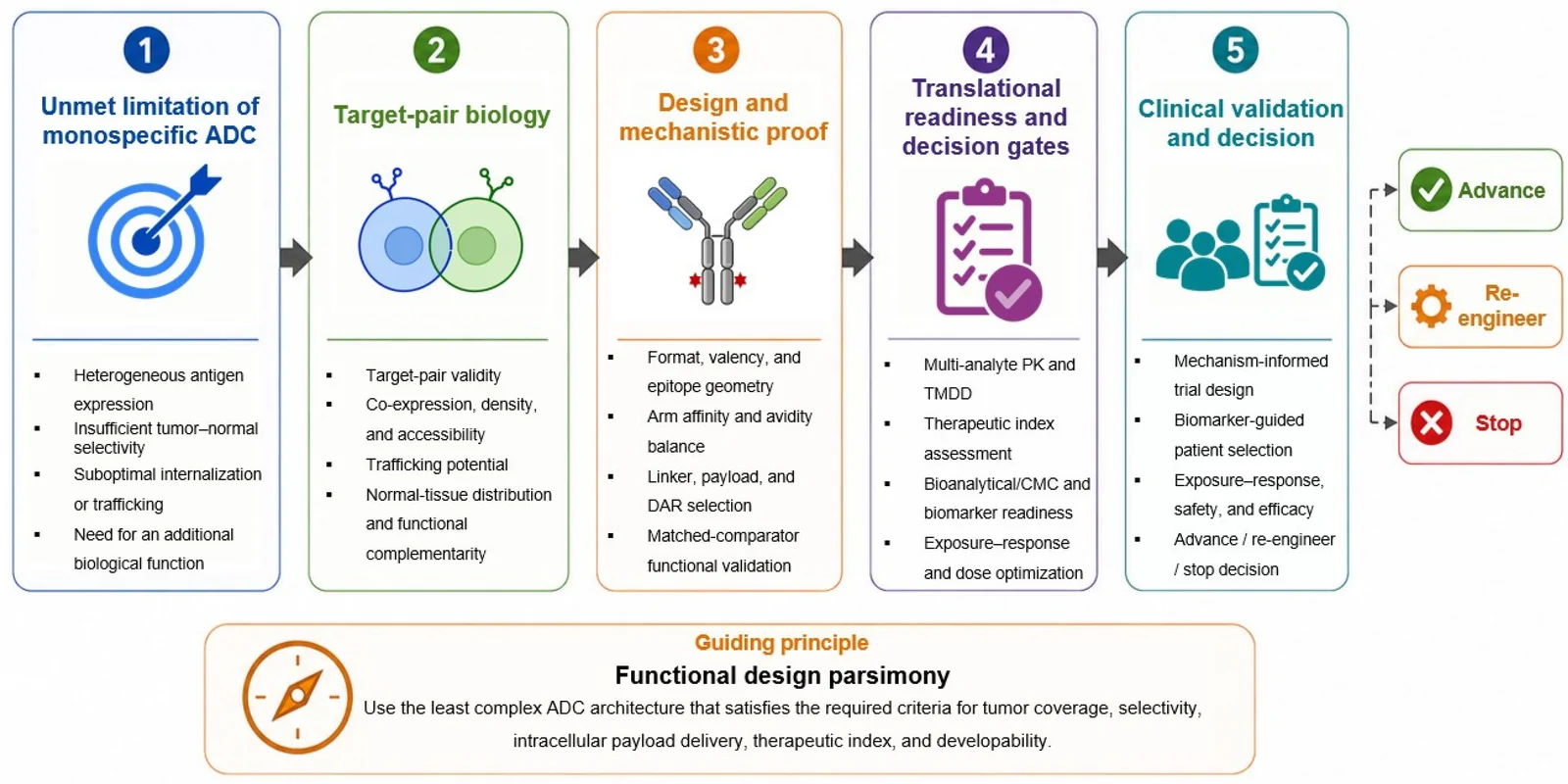
Proposed translational decision framework for the development of bispecific antibody–drug conjugates. The framework begins with a defined limitation of a monospecific ADC and proceeds sequentially through target-pair biology, molecular design and mechanistic proof, translational readiness, and early clinical validation. Target-pair assessment includes co-expression, antigen density and accessibility, normal-tissue distribution, and receptor-trafficking characteristics. Molecular design integrates antibody format and valency, epitope geometry, arm affinity and avidity, linker–payload properties, and drug-to-antibody ratio (DAR), with the incremental value of bispecificity evaluated against appropriately matched monospecific comparators. Translational readiness includes multi-analyte pharmacokinetics, target-mediated drug disposition (TMDD), therapeutic-index assessment, bioanalytical and chemistry, manufacturing, and controls (CMC) readiness, biomarker feasibility, and exposure–response relationships. Early clinical validation should assess mechanism-informed trial design, biomarker-defined activity, exposure–response relationships, safety, efficacy, and mechanisms of resistance. Evidence generated across these stages informs three potential development decisions: advance, when the predefined biological and translational criteria are met; re-engineer, when a potentially correctable limitation is identified and the candidate returns to design or optimization; or stop, when a major biological, pharmacological, safety, or developability liability is not adequately addressable. Functional design parsimony serves as the guiding principle throughout the framework, favoring the least complex ADC architecture that satisfies the required criteria for tumor coverage, selectivity, intracellular payload delivery, therapeutic index, and developability. Arrows indicate the intended progression through development and, where applicable, feedback from re-engineering to earlier design stages. The framework is intended as a structured decision tool rather than a universally validated numerical scoring system.

The clinical evidence and translational bottlenecks summarized above motivate a domain-based framework for deciding whether a bsADC is sufficiently robust for continued development. Figure 2 provides an overview of the proposed sequence from target-pair validation and mechanistic proof through translational readiness, clinical validation, and advance/re-engineer/stop decisions. The following subsections define the evidence expected within each development domain and the considerations that should inform progression.

### 6.1. Target-Pair Validity and Biomarker Readiness

The first requirement is to establish that the selected target pair occurs in the intended patient population in a pattern compatible with the proposed mechanism. Independent evidence that target A and target B are each expressed in a tumor type is not sufficient. Their prevalence, cellular co-expression, surface density, spatial distribution, intratumoral heterogeneity, and variation across primary and metastatic lesions should be characterized at the protein level whenever feasible. Evidence from dual-antigen programs and spatial tissue analyses supports evaluating co-expression, antigen density, and spatial organization at the target-pair level [11,12,27,29].

The relevant expression pattern differs according to the design strategy. For a coverage-oriented bsADC, the biological value of the second antigen lies in expanding the fraction of malignant cells that can be engaged beyond that achieved by either target alone. The relevant population is therefore the union of target A-positive and target B-positive cells. By contrast, a co-expression-dependent molecule relies on the intersection of the two antigen-positive populations and requires both targets to be present on the same cell at densities compatible with simultaneous engagement or cross-arm avidity.

This distinction cannot always be resolved using conventional bulk assays. A tumor may be classified as positive for both antigens while containing spatially separate target A-positive and target B-positive populations. Such a pattern may support coverage-oriented targeting but not an avidity-dependent strategy. Conversely, optical co-localization at the cellular or subcellular level does not prove that two epitopes are positioned within the nanometer-scale geometric constraints required for simultaneous engagement by a single bsADC molecule. Multiplex immunofluorescence, quantitative digital pathology, flow cytometry, spatial proteomics, and single-cell analyses are valuable for target-pair discovery and mapping, whereas productive cis-binding should be confirmed with molecular-format-specific binding and functional assays. The EpCAM×CLDN3, P-cadherin×CDH17, and CD33×CD7 programs illustrate the importance of defining same-cell co-expression rather than relying only on the prevalence of individual targets [8,11,29].

Target expression should also be examined in clinically representative specimens. Prior therapy, disease progression, metastatic site, and previous exposure to antigen-directed treatments may alter target abundance or enrich resistant subpopulations. Analyses restricted to treatment-naïve primary tumors may therefore overestimate the eligible population for an early clinical trial conducted in heavily pretreated metastatic disease.

Normal-tissue expression requires equally careful evaluation. The two antigens should be assessed independently and jointly, with attention to cell type, subcellular localization, vascular accessibility, density, and internalization. An antigen confined to an inaccessible epithelial surface may carry a different risk from one exposed to the systemic circulation. For co-expression-dependent designs, limited overlap of the two targets in normal tissue may support a wider therapeutic window, but this advantage should be demonstrated experimentally rather than inferred from separate expression atlases.

Additional target-related factors include antigen shedding, soluble target concentrations, receptor turnover, recycling, and physiological function. Soluble antigen may bind one arm, alter systemic disposition, or generate circulating complexes. A rapidly internalizing normal-tissue target may create a greater toxicity risk than an abundant but poorly accessible antigen. These properties should inform target-pair selection before extensive optimization of antibody format and linker–payload chemistry.

The biomarker strategy should be considered at the same stage as target validation. A highly sophisticated dual-expression hypothesis has limited clinical utility if it cannot be translated into a robust assay using routinely available tumor specimens. Discovery-stage spatial methods can define co-expression and heterogeneity, but clinical deployment may require standardized workflows such as chromogenic immunohistochemistry, validated digital pathology, or dual-color in situ assays that can be performed reproducibly in routine laboratories. Early development should therefore address specimen requirements, archival versus fresh tissue, pre-analytical variability, assay reproducibility, and whether target expression is best represented by separate cutoffs, a composite dual-expression score, or a spatial co-expression algorithm. The assay used in the clinical trial should reflect the intended mechanism rather than simply measure each antigen independently.

### 6.2. Functional Validation of Bispecificity

After the target pair has been established, the central preclinical question is whether dual recognition provides an advantage over an appropriate monospecific alternative. A bsADC may appear highly active because of its payload while deriving little incremental value from its second binding arm. Experimental comparisons should therefore be designed to isolate the contribution of bispecificity. Experimental bsADC and biparatopic studies likewise show that target density, avidity, receptor trafficking, and payload context can materially alter functional performance [10,11,12,13,24,28,30].

A useful model framework includes target A-positive/target B-negative, target A-negative/target B-positive, dual-positive, and double-negative cells. These models should cover clinically plausible antigen densities rather than relying exclusively on engineered overexpression. Isogenic systems are particularly useful for establishing causality because non-isogenic cell lines may differ in proliferation rate, efflux transporters, lysosomal protease activity, apoptotic threshold, and other determinants of ADC sensitivity. Findings should nevertheless be confirmed in naturally expressing tumor cells, organoids, or patient-derived models to preserve biological relevance.

The expected activity pattern should correspond to the intended design. A coverage-oriented bsADC should retain meaningful binding and cytotoxic activity in both single-positive populations and should perform consistently in heterogeneous cell mixtures. A co-expression-dependent construct should demonstrate a pre-specified and reproducible separation between dual-positive and single-positive cells in binding, internalization, payload delivery, or cytotoxicity. No universal two- or three-fold potency threshold can be applied across target pairs, payload classes, and assay formats; the criterion should instead exceed assay variability and be justified prospectively by receptor density, exposure, and the intended clinical mechanism. A trafficking-assisted molecule should show that the second target increases productive internalization, lysosomal delivery, or intracellular payload release relative to a target-matched monospecific ADC [10].

Comparator selection is critical. Depending on the mechanistic claim, informative controls may include the unconjugated bispecific antibody, parental monoclonal antibodies, payload-matched monospecific ADCs, a combination of the two monospecific ADCs, a nonbinding control ADC, and the unconjugated payload. Linker, payload, conjugation site, Fc configuration, DAR, total payload-to-antibody stoichiometry, and exposure should be matched as closely as technically feasible. Otherwise, improved activity may reflect differences in drug loading, hydrophobicity, linker stability, or payload potency rather than an advantage created by the bispecific architecture.

Conjugation may alter affinity, geometry, stability, and internalization, and the effect can be arm specific in an asymmetric molecule. Payload attachment or local structural perturbation near one antigen-binding region could selectively weaken one arm and shift the circulating product toward functionally monospecific behavior. Binding to each antigen and simultaneous engagement should therefore be reconfirmed using the final conjugated molecule. Orthogonal approaches can include individual-arm affinity measurements by surface plasmon resonance or biolayer interferometry, sequential or bridging formats that test simultaneous dual-antigen engagement, and cell-based assays at defined antigen densities. For co-expression-dependent designs, the final bsADC should retain the intended preference for dual-positive cells after conjugation.

Internalization should also be distinguished from productive payload delivery. The conjugate must reach an intracellular compartment that supports linker cleavage or antibody degradation, generate an active payload or catabolite, and engage the relevant intracellular target. Lysosomal localization should therefore be complemented where possible by measurement of mechanism-relevant species such as intact conjugate, linker-cleavage products, released payload, and antibody-associated catabolites using ligand-binding, chromatographic, or mass-spectrometric methods. Time-dependent cytotoxicity and pharmacodynamic markers can then link receptor engagement to pharmacological activity.

Bystander killing should be evaluated separately from direct target-mediated cytotoxicity. Mixed co-cultures, three-dimensional spheroids, and mosaic tumors can determine whether released payload reaches adjacent antigen-low or antigen-negative cells. Membrane-permeable payloads may partially compensate for heterogeneous antigen expression, but they may also reduce the cellular precision achieved through co-expression-dependent binding [26]. The relative contributions of antibody-mediated targeting and payload diffusion should therefore be distinguished when interpreting activity in heterogeneous models.

For multifunctional bsADCs, the contributions of payload delivery, receptor blockade, immune-checkpoint inhibition, and Fc-mediated activity should be separated where possible. The preclinical characterization of DB-1419, for example, incorporated dual-target binding, internalization, direct cytotoxicity, bystander activity, checkpoint inhibition, Fc-mediated effects, pharmacokinetics, and nonhuman-primate safety [30]. Such breadth strengthens the translational argument when each proposed mechanism is linked to an appropriate comparator and pharmacodynamic marker.

### 6.3. Translational Models and PK/PD Integration

In vivo development should use models selected to test the intended value of bispecificity rather than merely to demonstrate tumor growth inhibition. A dual-positive xenograft with high expression of both targets may establish proof of activity but provides limited information regarding heterogeneity, patient selection, and therapeutic index. More informative panels include tumors with different combinations and densities of the two antigens, together with heterogeneous models containing distinct single-positive and dual-positive populations. ADC and bispecific pharmacology studies further support integrating antigen-dependent exposure, TMDD, tumor penetration, and model-informed translation rather than relying on tumor-growth inhibition alone [3,25,41,42,48,49,50,51].

Cell line-derived xenografts remain useful for controlled comparisons, whereas patient-derived xenografts, organoids, and organoid-derived xenografts may better preserve tumor architecture, clinically relevant antigen heterogeneity, and resistance states. Three-dimensional models are particularly useful because receptor accessibility, antibody penetration, and payload diffusion differ substantially from two-dimensional culture. However, conventional CDX and PDX studies in immunocompromised mice can underestimate target-mediated clearance and normal-tissue toxicity when the clinical antibody does not cross-react with the corresponding murine targets. In vivo imaging or dual-label approaches may further distinguish total antibody accumulation from intact ADC distribution and released-payload exposure, but model selection should explicitly acknowledge which human target-mediated sinks are absent.

The required model depends on the design logic. Coverage-oriented molecules should be tested in tumors containing separate target A-positive and target B-positive compartments. Co-expression-dependent constructs should be compared across dual-positive and relevant single-positive tumors, and their selectivity should remain evident at clinically plausible exposures. Trafficking-assisted bsADCs should demonstrate improved intracellular payload delivery rather than merely higher total tumor antibody accumulation.

Efficacy assessment should extend beyond short-term tumor growth inhibition. Depth and durability of response, complete-regression frequency, time to regrowth, activity in established tumors, and response following retreatment or treatment interruption provide more informative evidence. Recurrent tumors may be examined for antigen loss, altered target ratios, changes in trafficking, and payload resistance to determine whether dual targeting delays biological escape or simply increases initial cytotoxic exposure.

The pharmacokinetic strategy will typically need to distinguish total antibody, conjugated or intact ADC, and unconjugated payload, depending on the molecular design and linker–payload system. Additional measurements may include DAR distribution, released catabolites, soluble target complexes, and anti-drug antibodies (ADAs). Quantitative interpretation should consider analyte-specific AUC, Cmax, trough concentration, intact-to-total antibody ratios, free-payload exposure, and time-dependent changes in DAR where informative. These metrics provide distinct information regarding carrier exposure, active delivery-form exposure, deconjugation, systemic payload toxicity, and the potential effect of dose fractionation.

Bispecificity may introduce more complex target-mediated drug disposition because either antigen can contribute a parallel pathway of tissue binding, internalization, and clearance. The relative contribution of each pathway may vary with dose as one or both target pools become partially saturated, and soluble antigen can add an additional circulating sink. Dual-positive tissues may also display avidity-dependent retention that is not predictable from the disposition of either parental antibody. Pharmacokinetic characterization should therefore be conducted using the final conjugated molecule and, where feasible, should evaluate dose dependence in clearance and exposure rather than extrapolating from the unconjugated bispecific antibody.

Plasma exposure alone is not a sufficient surrogate for tumor payload delivery. Intratumoral exposure depends on vascular permeability, tissue penetration, antigen density, association and dissociation kinetics, receptor turnover, internalization, linker processing, and payload efflux. High-affinity binding to abundant perivascular antigen can create a binding-site barrier, increasing peripheral retention while reducing deeper penetration. Studies of conventional ADCs have shown that tumor payload concentrations may correlate more closely with antigen expression and intracellular processing than with total antibody exposure alone [49].

Mechanistic PK/PD or quantitative systems pharmacology models can integrate systemic exposure, association and dissociation rates, antigen density, receptor turnover, internalization, target-mediated degradation, tumor distribution, payload release, tissue-specific clearance, and tumor response. Such models have been developed for conventional ADCs to support human exposure and efficacy predictions across different antigen-expression states [50,51]. For bsADCs, the same framework can be extended to incorporate two target-binding and internalization pathways and their potential saturation. The purpose of such modeling is not to replace experimental evidence but to identify the parameters most likely to determine dose, schedule, efficacy, and toxicity and to prioritize measurements that reduce uncertainty.

### 6.4. Nonclinical Safety and Species Relevance

The safety profile of a bsADC may be driven by either target, the bispecific scaffold, Fc-mediated activity, linker instability, released payload, or interactions among these factors. Safety assessment should therefore distinguish on-target/off-tumor toxicity from target-independent payload toxicity and from liabilities introduced by the bispecific format. Established ADC safety frameworks and tissue cross-reactivity studies support this integrated evaluation of target-, payload-, and species-dependent risk [7,18,40,52].

Normal-tissue risk should be evaluated for both antigens. For a coverage-oriented bsADC, binding to either target may be sufficient to produce toxicity. For a co-expression-dependent construct, the key question is whether avidity-enhanced binding and internalization occur in any normal cell population. Rare dual-positive normal cells may not be evident in separate bulk-expression datasets, making cell-type localization and tissue cross-reactivity particularly important.

Selection of a pharmacologically relevant toxicology species may be difficult when two targets are involved. Ideally, the test species should support binding of both arms, preserve the intended relative affinity and avidity relationship, and reproduce relevant human tissue-expression patterns. Nominal cross-reactivity alone is insufficient: even modest human–animal differences in affinity, receptor density, epitope accessibility, or internalization can change TMDD and toxicologic exposure. Cross-reactivity with only one target may characterize some risks but cannot fully assess toxicity arising from simultaneous engagement. Tissue cross-reactivity studies can assist species selection but should be interpreted together with quantitative binding, expression biology, functional pharmacology, and systemic toxicology [52].

When no single species is relevant for both targets, an integrated approach may be necessary. This may include cross-reactive surrogate molecules, separate evaluation of the individual binding arms, human primary-cell assays, organotypic systems, and studies of the clinical ADC in a species relevant to payload exposure. Surrogates can be informative but do not necessarily reproduce the human molecule’s epitope geometry, expression level, binding kinetics, valency, conjugation, or Fc activity; these differences should be explicitly considered when interpreting safety margins.

Toxicokinetic measurements should relate adverse findings to both total and conjugated exposure and, where relevant, to free payload or active catabolites. Payload-associated hematologic, hepatic, pulmonary, gastrointestinal, neurologic, or ocular effects should be interpreted in the context of payload chemistry, intracellular catabolite formation, linker stability, tissue distribution, and target expression rather than presented only as aggregate organ-level findings. Repeat-dose studies are generally more informative than single-dose tolerability because accumulation, delayed toxicity, deconjugation, and reversibility may only become evident after repeated administration [40].

Multifunctional constructs may also require evaluation of cytokine release, immune-cell activation, checkpoint-related effects, and Fc-mediated pharmacology. Immunogenicity risk should be considered during candidate selection, particularly when the molecule contains non-native junctions, substantial sequence engineering, aggregation-prone regions, or conjugation-induced structural changes. Where anti-drug antibodies are detected, domain-specific or epitope-mapping approaches can help distinguish responses to engineered junctions, individual binding domains, Fc regions, or conjugation-associated neo-epitopes. Nonclinical anti-drug antibody findings have limited ability to predict clinical immunogenicity, but they may interfere with exposure and interpretation of animal studies and should therefore be evaluated in context.

### 6.5. Manufacturability and Analytical Control

A bsADC should progress toward formal development only when both the bispecific antibody and the final conjugated product can be manufactured reproducibly at an appropriate scale. Engineered bispecific formats may be more susceptible than conventional IgGs to heavy-chain homodimerization, heavy-chain/light-chain mispairing, broader chain-scrambling species, low expression, aggregation, fragmentation, molecular asymmetry, and instability. These liabilities have different structural consequences and may require distinct engineering and analytical controls. Published ADC manufacturing and bispecific developability studies likewise support early assessment of molecular stability, chain pairing, conjugation, and comparability [19,43,44,45,53].

Developability assessment should begin during lead selection. Relevant attributes include correct chain assembly, yield, purity, thermal and colloidal stability, nonspecific binding, self-interaction, aggregation propensity, viscosity, and stability under representative process and storage stresses. Comparative analyses have suggested that some engineered bispecific and fragment-based formats carry greater stability risks than conventional full-length antibodies, supporting early developability screening rather than postponing it until CMC development [43].

Conjugation may amplify pre-existing liabilities by increasing hydrophobicity, altering surface charge, modifying residues involved in binding or structural stability, and broadening product heterogeneity. The magnitude of this effect depends on payload chemistry, linker hydrophobicity, conjugation site, and DAR; site-specific conjugation and controlled DAR can improve consistency but do not eliminate the need for product-specific developability assessment. Antibody format, linker–payload chemistry, conjugation site, DAR, and formulation should therefore be co-optimized.

Manufacturability of the unconjugated bispecific antibody does not guarantee manufacturability of the final bsADC. Binding, aggregation, structural integrity, and stability should be reassessed after conjugation. In one bispecific-antibody case study, a molecule that appeared acceptable during discovery developed substantial precipitation during scale-up under agitation stress; sequence engineering was required to reduce surface hydrophobicity and improve conformational stability [53]. This illustrates why discovery engineering and CMC assessment should be connected before candidate nomination.

The analytical package should characterize molecular identity, chain pairing, purity, fragments, aggregates, charge and size variants, DAR distribution, conjugation-site occupancy, free payload, and linker stability. Representative orthogonal methods can include size-exclusion chromatography for aggregates, capillary electrophoresis for size variants, hydrophobic interaction chromatography or mass spectrometry for DAR-related heterogeneity, and surface plasmon resonance or biolayer interferometry for arm-specific and simultaneous target engagement. Binding to each antigen should be assessed individually together with a functional assay reflecting the intended mechanism. Acceptance criteria should remain product- and stage-specific rather than being inferred from a universal threshold, because the functional consequence of a given variant depends on architecture and mechanism.

Not every mechanistic assay needs to become a formal release test. However, critical quality attributes should be linked to functional consequences, and the control strategy should distinguish variants that merely alter analytical appearance from those that affect binding, pharmacokinetics, potency, or safety. For bsADCs whose mechanism depends on coordinated dual recognition, a dual-antigen functional assay or an appropriately qualified surrogate should be considered during lot release, stability, or comparability assessment. Reference standards and comparability plans should be established early because changes in cell line, scale, purification, conjugation, or formulation may alter the balance between the two binding arms.

### 6.6. A Practical Preclinical Go/No-Go Framework

The decision to advance a bsADC should be based on convergent evidence across five domains rather than exceptional performance in a single efficacy model. This domain-based approach is consistent with current nonclinical, CMC, clinical-pharmacology, and dose-optimization guidance, although specific thresholds remain product dependent [18,19,45,46,47,48].

First, target-pair validity and biomarker readiness require evidence that the two antigens occur in the intended patient population in a distribution compatible with the proposed mechanism, together with a clinically deployable method for identifying that population.

Second, functional value of bispecificity requires a reproducible advantage over relevant payload-matched monospecific comparators. The advantage should correspond to the intended design: broader coverage, co-expression-dependent selectivity, enhanced intracellular trafficking, or a clearly demonstrated additional biological function.

Third, translational pharmacology requires that this advantage persists in representative three-dimensional and in vivo models at clinically plausible exposures. PK, tumor distribution, payload release, exposure–response relationships, and durability should support a feasible human dose and schedule.

Fourth, therapeutic-index feasibility requires a credible separation between efficacy-associated exposure and on-target normal-tissue effects, payload-related toxicity, or liabilities introduced by dual engagement. Limitations of the toxicology species or surrogate strategy should be understood before first-in-human dosing.

Fifth, developability and analytical control require scalable production, acceptable molecular stability, controlled conjugation, mechanism-relevant potency assays, and a comparability strategy capable of supporting clinical manufacturing.

Strength in one domain cannot necessarily compensate for a critical weakness in another. A practical hierarchy is to first exclude target-pair and normal-tissue liabilities that make a therapeutic window implausible, then confirm mechanism-specific functional value over matched comparators, and only thereafter invest heavily in translational PK, formal toxicology, and scale-up. Exceptional xenograft efficacy does not overcome an implausible normal-tissue risk, and favorable manufacturability does not justify a molecule whose second binding arm provides no measurable biological advantage. Quantitative go/no-go criteria should be prespecified where a validated relationship exists, but universal cutoffs for affinity, fold-selectivity, IC50 shift, target occupancy, or exposure separation are not currently established across target pairs, payload classes, and molecular formats. The relative threshold in each domain therefore remains product-, indication-, and assay-specific.

These domains are proposed as decision gates rather than as a formally validated numerical scoring system. The evidence requirements and advancement criteria for preclinical-to-clinical translation are summarized in Table 3. Figure 2 integrates these domains into a sequential development framework spanning target-pair validation, functional characterization, translational pharmacology, safety, biomarker readiness, and CMC development. Together, these tools may help distinguish bsADCs that are merely active in experimental models from those with a credible path toward clinical validation.

## 7. Conclusions

Bispecific antibody–drug conjugates are designed to address key limitations of conventional ADCs, including heterogeneous antigen expression, insufficient tumor selectivity, and inefficient intracellular trafficking. Depending on their design, bsADCs may broaden tumor-cell coverage, favor dual-positive cells, enhance internalization, or combine payload delivery with an additional biological function. However, these advantages are not inherent to bispecificity and depend on the integrated effects of target-pair biology, antibody architecture, linker–payload properties, pharmacokinetics, and normal-tissue distribution.

Preclinical activity alone is insufficient to establish translational superiority. The incremental value of the second binding arm should be demonstrated under clinically relevant antigen-expression conditions and against appropriately matched monospecific ADC comparators. Target-pair validity, functional benefit, therapeutic-index feasibility, manufacturability, and biomarker readiness should therefore be evaluated as an integrated development package.

Clinical studies have confirmed the feasibility of selected dual-binding ADC architectures, but broader class-level validation remains premature. Izalontamab brengitecan currently provides the most mature evidence, while JSKN016 and TQB2102 have generated encouraging clinical signals. In contrast, MEDI4276 illustrates that enhanced receptor engagement and internalization do not necessarily produce an adequate therapeutic window. These experiences indicate that clinical performance is determined by the complete antibody–linker–payload configuration rather than by bispecificity alone.

The independent clinical contribution of bispecificity also remains unresolved because closely matched monospecific comparators are generally unavailable. Superiority over chemotherapy validates the complete drug product but does not determine whether benefit derives from dual recognition, enhanced trafficking, bystander killing, or payload sensitivity. Prospective biomarker development and exposure–response analyses will therefore be essential to define the populations and mechanisms underlying clinical benefit.

Future programs should begin with a clearly defined limitation of monospecific ADC therapy and select the target pair, molecular format, linker, payload, DAR, and dosing strategy to address that problem. CMC, analytical development, biomarker strategy, and clinical pharmacology should proceed in parallel.

We propose functional design parsimony as a guiding principle for bsADC development: the least complex architecture capable of achieving the required tumor coverage, selectivity, intracellular delivery, and therapeutic index should be preferred. Added valency, bispecificity, multifunctionality, or payload complexity should be retained only when it produces measurable incremental value that remains compatible with pharmacokinetics, safety, biomarker implementation, analytical control, and manufacturing. Bispecificity should therefore be viewed not as the therapeutic objective itself, but as an engineering solution whose incremental value must be demonstrated against simpler alternatives.

## Figures and Tables

**Table 1:** Functional design strategies of bispecific antibody–drug conjugates.

Design Category	Core Mechanism and Intended Advantage	Representative Examples	Principal Development Risk	Ref.
Coverage-oriented targeting	Independent binding to either target broadens payload delivery across heterogeneous tumor-cell populations	SORT1×HER2-DXd	Additional normal-tissue sinks and TMDD; limited incremental value when bystander killing already compensates for heterogeneity	[24]
Co-expression-dependent targeting	Cross-arm avidity favors dual-positive tumor cells over single-positive normal cells	EpCAM×CLDN3-DXd; P-cadherin×CDH17-MMAE; CD33×CD7 ADC	Requires stable same-cell co-expression; loss of either antigen may permit escape	[11,12,29]
Trafficking-assisted targeting	A second receptor promotes productive internalization or lysosomal routing of the target-bound complex	HER2×CD63; EphA2×ALCAM	Benefit depends on antigen density, affinity, geometry, and receptor trafficking	[10,28]
Multifunctional targeting	Payload delivery is combined with an additional immune or signaling function	DB-1419, B7-H3×PD-L1 bsADC	Multiple mechanisms complicate efficacy attribution, safety assessment, and potency testing	[30]
Biparatopic targeting ^1^	Binding to two epitopes on one antigen alters receptor organization through intra- or intermolecular engagement, internalization, and trafficking	HER2 biparatopic ADCs; MEDI4276; TQB2102	Benefit depends on epitope geometry, valency, linker–payload design, DAR, and therapeutic-index separation	[13,14,31]

^1^ Biparatopic ADCs recognize two non-overlapping epitopes on the same antigen and are included as mechanistically related comparators rather than dual-antigen bsADCs. Abbreviations: ADC, antibody–drug conjugate; bsADC, bispecific antibody–drug conjugate; DXd, deruxtecan-related topoisomerase I inhibitor payload; MMAE, monomethyl auristatin E.

**Table 2:** Clinical landscape of bispecific and biparatopic antibody–drug conjugates.

Agent	Target/Design	Payload	Clinical Evidence and Interpretation	Ref.
Izalontamab brengitecan (BL-B01D1/BMS-986507)	EGFR×HER3 bsADC; 2+2 IgG-(scFv)2	Topoisomerase I inhibitor (Ed-04); DAR ~8	Activity has been reported across multiple solid tumors, with randomized phase III evidence. It is the most mature dual-antigen bsADC, although the incremental contribution of bispecificity remains unresolved.	[16,33,34,35,36,37,38]
JSKN016	TROP2×HER3 bsADC; site-specific glycan conjugation	Topoisomerase I inhibitor; DAR 4	Early activity has been reported in advanced breast cancer, and randomized phase III development is ongoing. Comparative and biomarker data remain immature.	[39]
TQB2102	HER2 ECD2×ECD4 biparatopic ADC	Deuterated DXd-related topoisomerase I inhibitor; DAR ~5.8–6.0	Phase I activity supports the clinical feasibility of biparatopic HER2 targeting, although the evidence remains nonrandomized.	[31]
MEDI4276	HER2 ECD2×ECD4 tetravalent biparatopic ADC	AZ13599185 tubulysin derivative; site-specific DAR ~4	Phase I activity was constrained by gastrointestinal and hepatic toxicity, highlighting the integrated effects of architecture, payload, and exposure on therapeutic index.	[14,15]
DB-1419	B7-H3×PD-L1 multifunctional bsADC	Topoisomerase I inhibitor	Preclinical data support payload delivery, bystander activity, checkpoint blockade, and Fc-mediated effects; the clinical contribution of these mechanisms remains unconfirmed.	[30]

Abbreviations: ADC, antibody–drug conjugate; bsADC, bispecific antibody–drug conjugate; EGFR, epidermal growth factor receptor; HER2/3, human epidermal growth factor receptor 2/3; PD-L1, programmed death-ligand 1; TROP2, trophoblast cell-surface antigen 2. Note: Biparatopic ADCs are included as mechanistically related comparators. Development status reflects publicly available information as of 29 July 2026.

**Table 3:** Proposed evidence framework for preclinical-to-clinical translation of bsADCs.

Translational Domain	Evidence Required	Advancement Criterion	Ref.
Target-pair validity and biomarker readiness	Same-cell co-expression, antigen density, spatial heterogeneity, normal-tissue distribution, and a clinically applicable assay	A clinically relevant target pattern and reproducibly identifiable patient population	[11,12,29]
Functional value of bispecificity	Binding, internalization, payload delivery, and cytotoxicity compared with payload-matched monospecific ADCs	A pre-specified, mechanism-consistent advantage beyond assay variability; no universal fold threshold	[10,13,28]
Translational pharmacology	Representative heterogeneous models, multi-analyte PK, tumor distribution, stability, AUC/Cmax/trough and exposure–response analysis	Durable activity at a clinically feasible dose and schedule	[15,49]
Therapeutic-index feasibility	Normal-tissue expression, cross-reactivity, repeat-dose toxicokinetics, and payload-related toxicity	Product-specific separation between active and toxic exposures supported by relevant PK/TK analytes	[40,52]
Developability and analytical control	Chain pairing, purity, stability, DAR, linker integrity, arm-specific binding, simultaneous dual-target engagement, and functional potency	Reproducible manufacturing and retained activity across lots and scales	[43,53]

Abbreviations: ADC, antibody–drug conjugate; bsADC, bispecific antibody–drug conjugate; DAR, drug-to-antibody ratio; PK, pharmacokinetics. Note: this framework is proposed by the authors and is not a formally validated regulatory scoring system.

## Data Availability

No new data were created or analyzed in this study. Data sharing is not applicable to this article.

## Abbreviations

The following abbreviations are used in this manuscript:

ADC	antibody–drug conjugate
bsADC	bispecific antibody–drug conjugate
CMC	chemistry, manufacturing, and controls
DAR	drug-to-antibody ratio
EGFR	epidermal growth factor receptor
HER2	human epidermal growth factor receptor 2
HER3	human epidermal growth factor receptor 3
MMAE	monomethyl auristatin E
PD-L1	programmed death-ligand 1
PK	pharmacokinetics
NSCLC	non-small-cell lung cancer
SCLC	small-cell lung cancer
TNBC	triple-negative breast cancer
TROP2	trophoblast cell-surface antigen 2
ALCAM	activated leukocyte cell adhesion molecule
CDH17	cadherin 17
CLDN3	claudin 3
DXd	deruxtecan-derived topoisomerase I inhibitor payload
EpCAM	epithelial cell adhesion molecule
EphA2	ephrin type-A receptor 2
Fc	fragment crystallizable region
PD	pharmacodynamics
SORT1	sortilin 1
